# Intact cluster and chordate-like expression of ParaHox genes in a sea star

**DOI:** 10.1186/1741-7007-11-68

**Published:** 2013-06-27

**Authors:** Rossella Annunziata, Pedro Martinez, Maria Ina Arnone

**Affiliations:** 1 Stazione Zoologica Anton Dohrn di Napoli, Cellular and Developmental Biology, Villa Comunale, 80121 Napoli, Italy; 2 Departament de Genètica, Universitat de Barcelona, Av. Diagonal, 643, 08028 Barcelona, Spain; 3 Institució Catalana de Recerca i Estudis Avançats (ICREA), Passeig Lluís Companys, 23 08010, Barcelona, Spain

**Keywords:** Patiria miniata, Asteroid, ParaHox, Cluster, Gsx, Xlox, Cdx, Colinearity, Gut

## Abstract

**Background:**

The ParaHox genes are thought to be major players in patterning the gut of several bilaterian taxa. Though this is a fundamental role that these transcription factors play, their activities are not limited to the endoderm and extend to both ectodermal and mesodermal tissues. Three genes compose the ParaHox group: *Gsx*, *Xlox* and *Cdx*. In some taxa (mostly chordates but to some degree also in protostomes) the three genes are arranged into a genomic cluster, in a similar fashion to what has been shown for the better-known Hox genes. Sea urchins possess the full complement of ParaHox genes but they are all dispersed throughout the genome, an arrangement that, perhaps, represented the primitive condition for all echinoderms. In order to understand the evolutionary history of this group of genes we cloned and characterized all ParaHox genes, studied their expression patterns and identified their genomic loci in a member of an earlier branching group of echinoderms, the asteroid *Patiria miniata*.

**Results:**

We identified the three ParaHox orthologs in the genome of *P. miniata*. While one of them, *PmGsx* is provided as maternal message, with no zygotic activation afterwards, the other two, *PmLox* and *PmCdx* are expressed during embryogenesis, within restricted domains of both endoderm and ectoderm. Screening of a *Patiria* bacterial artificial chromosome (BAC) library led to the identification of a clone containing the three genes. The transcriptional directions of *PmGsx* and *PmLox* are opposed to that of the *PmCdx* gene within the cluster.

**Conclusions:**

The identification of *P. miniata* ParaHox genes has revealed the fact that these genes are clustered in the genome, in contrast to what has been reported for echinoids. Since the presence of an intact cluster, or at least a partial cluster, has been reported in chordates and polychaetes respectively, it becomes clear that within echinoderms, sea urchins have modified the original bilaterian arrangement. Moreover, the sea star ParaHox domains of expression show chordate-like features not found in the sea urchin, confirming that the dynamics of gene expression for the respective genes and their putative regulatory interactions have clearly changed over evolutionary time within the echinoid lineage.

## Background

Homeobox-containing genes regulate many aspects of development in Bilateria. Based on sequence similarities, the presence of specific protein motifs, genomic arrangement and other characteristics these genes have been classified into several families. Among the best-characterized families are the Hox and the ParaHox. While the Hox family is best characterized in segmented animals (arthropods and vertebrata), where they play patterning roles in the three germ layers, little is known about the role of ParaHox in most taxa, although it is agreed that they serve an integral role in endoderm development.

A key property of Hox genes in most Bilateria is their genomic arrangement in clusters. The position of different genes within the cluster is related to their relative domains of expression along the major body axis. This property, called spatial colinearity is observed in most animals, even in those with a clearly derived morphology (that is, echinoderms [1]). Some taxa also exhibit a different form of colinear expression of genes, temporal colinearity, in which genes located in different positions within the cluster are activated progressively during development. While temporal colinearity seems to be strictly dependent on the presence of an intact cluster, spatial colinearity seems to be more permissive with cluster breaks. In fact, there are cases of extreme cluster disintegration, for instance in larvaceans [2] or acoelomorphs [3], where the relative spatial domains are still well conserved (with respect to the genomic locations that their clustered paralogs exhibit in other groups). This form of colinearity, in the absence of a genomic cluster, has been named trans-colinearity [4].

The general features exhibited by the ParaHox genes in animals are less clear. While many ParaHox genes have been identified in different animal groups, little is known about their genomic arrangements and developmental roles. A general agreement is that the ParaHox genes, *Gsx*, *Xlox* and *Cdx*, are an ancient sister group to the Hox genes. Both groups probably evolved from a primitive ProtoHox cluster that was duplicated, giving rise to the Hox and ParaHox clusters [5]. Surprisingly, while the presence of a Hox cluster has been demonstrated in many animal groups, ‘intact’ ParaHox clusters are only known from Chordata. It is presently unclear whether there are complete ParaHox clusters in any protostome group (though a partially intact cluster has been described in *Platynereis dumerilii*: [6]) or in any non-chordate deuterostome. It is interesting to note that in some groups, such as sea urchins, the absence of cluster organization has not prevented the ParaHox genes from showing signs of correlative expression (both spatial and temporal [7]). Moreover, in that system, two of the genes (*Xlox* and *Cdx*) are clearly interlinked within a gene regulatory network that controls endoderm regionalization and exhibit mutual regulatory interactions [8]. Whether this property is a product of an older (evolutionary) cluster association is not yet known.

The role of bilaterian ParaHox genes (with the exception of *Gsx* that is almost entirely expressed in the central nervous system (CNS)) seems to relate to both the CNS and endoderm patterning processes. The orthologs of the gene *Gsh*, the most ‘anterior’ of the ParaHox genes, are expressed in different domains of the bilaterian nervous system. A glance at the expression of the three ParaHox genes within the Bilateria shows their broad commonalities, most probably a reflection of ancestral roles. For instance, the mice *Gsh1* and *Gsh2* paralogs are mostly active in the developing brain [9,10]. The related gene in amphioxus, *AmphiGsx*, appears first in four cells in the neural tube at the level of somite five, and later only in the cerebral vesicle [11]; likewise, in the ascidian *Ciona intestinalis*, its ortholog is expressed in the sensory vesicle [12]. In the sea urchin embryo the gene *SpGsx* is detected in a small ectodermal domain, probably neural [7]. Within the Protostomia, *Gsx* has been analyzed in a few phyla. In insects (where it receives the name *ind*) the gene is expressed in the intermediate columns of the CNS [13]. In nereid worms (*P. dumerilii* is the best studied example) *Gsx* is expressed in two domains, one in the CNS and another in the stomadeum plus some cell clusters of the posterior foregut and midgut [6]. Interestingly, in other polychaete worms, such as *Capitella teleta*, the gut domain has been lost [14]. Dual domains of expression, in the CNS and the gut, are similarly observed in the mollusk *Gibbula varia*[15]. Interestingly, in this last case it has been observed that after torsion, the domains of *Gsx* expression are localized to the mouth and foregut, somewhat reminiscent of the pattern seen in *Platynereis*. All these patterns observed in different bilaterian taxa suggest a primitive function of *Gsx* in patterning the CNS and the most anterior gut.

The central ParaHox gene, *Xlox*, has been also described in different groups of Bilateria. Strikingly, the gene seems to be lost from all insect genomes (not present in any of the genomes sequenced to date). In vertebrates, *Xlox* is expressed in both the developing gut [16,17] and the CNS [18]. In the chordate amphioxus, *AmphiXlox* is detected in the gut and in two cells of the neural tube [5]. Similar domains of expression (gut plus neural ectoderm) are described for the sea urchin *Xlox* ortholog [7,19]. In protostomes the patterns are, again, very similar. In the mollusk *Gibbula*, *Xlox* is expressed in the middle part of the digestive tract and in some neuroectodermal cells [15]. Also in the polychaetes *Nereis virens* and *P. dumerilii* this gene is expressed in the midgut and in several cells of the neuroectoderm [6,20]. In some other protostomian taxa the domains are more restricted. In the leech (*Helobdella triserialis*) it has been reported that *Xlox* is only expressed in the midgut [21] while in the platyhelminth *Schmidtea polichroa* the ortholog gene is only expressed in the nervous system [22]. We see again that *Xlox* seems to be dedicated to the patterning of the gut (primarily) and some areas of the CNS.

*Cdx* (or *caudal* in insects) is the most ‘posterior’ of the ParaHox genes. It has been cloned and studied in many taxa. Three mouse paralogs (*Cdx1*, *Cdx2* and *Cdx4*) are expressed in the posterior part of the gut and some areas of the CNS [23], domains that are shared by most vertebrates. The amphioxus ortholog, *AmphiCdx*, is also expressed in the posterior part of the gut and in the developing neural tube [5]. In the sea urchin, *SpCdx* is active in the hindgut [7]. Urochordates express *Cdx* in cells of the neural plate and the posterior gut [24,25], although, in at least one case (in *Herdmania curvata*), both domains appear at different phases of their life cycle [24]. In several arthropods, the caudal gene patterns the posterior end of the animal [26-28]. In polychaetes *Cdx* is expressed both in the posterior region, including the gut, in addition to areas of the CNS. The mollusk *Gibbula* expresses its *Cdx* gene in the posterior end of the digestive tract, whereas in the veliger larva, expression is localized specifically in the hindgut and rectum [15]. Here, again, it is demonstrated that the *Cdx* genes are involved, as observed for *Gsx* and *Xlox*, in the patterning of structures located within the endoderm (mostly the posterior gut) and the neuroectoderm.

The commonalities shown in the expression of ParaHox genes have prompted some investigators to propose an ancestral role for this group of genes in the patterning of endoderm [29,30] (although we know that the similarities extend to other tissues). An important issue remains to be clarified: how do these commonalities together with the taxon specificities, reflect the history of the original cluster in every clade. The problem is accentuated by the paucity of information related to the genomic organization of these genes in most phyla.

Previous analysis of a sea urchin cluster revealed that the echinoid ParaHox genes were dispersed in the genome. This finding correlates with the rearrangement that occurred within the Hox cluster in the sea urchin genome and represents a clearly derived condition for ParaHox genes. In order to understand the origin of this disruption of genomic links we decided to analyze the history of echinoderm ParaHox clusters and the roles of its genes. Here we present a detailed investigation of ParaHox gene expression and genome organization in a representative of an earlier branching group of echinoderms, the sea star *P. miniata*. Asteroids and echinoids diverged around 480 million years ago (Mya) [31]. We find interesting differences in the genomic organization of the ParaHox genes in both groups. While, as previously shown, echinoids have all genes dispersed in the genome [7], here, in asteroids we find that all of them form a single, compact, cluster. Moreover, we find partial conservation and some remarkable divergent features in ParaHox gene expression patterns when compared with those of chordates and sea urchins.

## Results and discussion

### Identification of the *Patiria miniata* ParaHox genes: *PmGsx*, *PmLox*, and *PmCdx*

**Figure 1:**
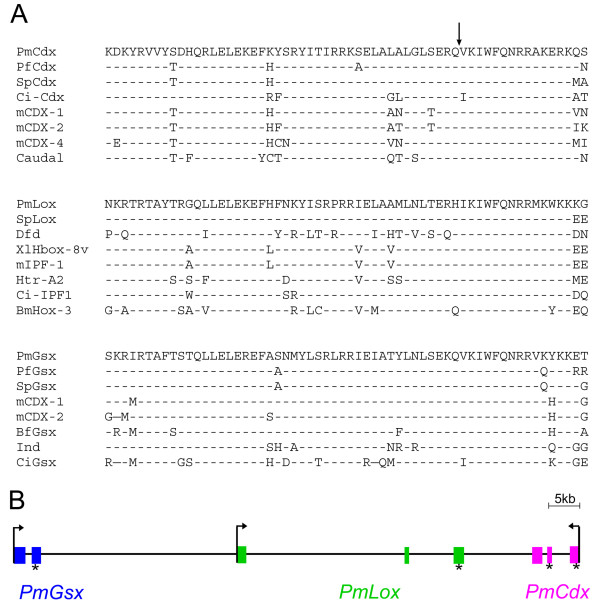
**Identification of the sea star *Patiria miniata *ParaHox orthologs. (A)** Alignment of the homeodomains of the three *P. miniata* ParaHox genes with representative invertebrate and vertebrate orthologs. Broken lines indicate amino acid identities; the position of *PmCdx* intron within the homeodomain is indicated by a vertical arrow. The names of the species are: Sp (*Strongylocentrotus purpuratus*); Pf (*Ptychodera flava*); Ci (*Ciona intestinalis*); m (*Mus musculus*); Dfd (*Drosophila melanogaster*); Xl (*Xenopus laevis*); Htr (*Helobdella triserialis*); Bf (*Branchiostoma floridae*). *ind* and *caudal* are the *Drosophila melanogaster*vorthologs of *Gsx* and *Cdx* respectively. **(B)** The genomic organization of *P. miniata* ParaHox genes is represented in scale showing separate exons for each gene. Asterisks indicate the homeobox in each gene. kb: kilobases.

Cloning of *P. miniata* ParaHox genes was carried out by a combination of PCR methodology with 3′rapid amplification of cDNA ends (3′RACE) and sequencing of bacterial artificial chromosome (BAC) genomic clones (for details see Methods). The orthology assignment of the three ParaHox genes was determined by phylogenetic analysis, as provided in Additional file 1: Figure S1. The alignment of ParaHox homeodomains with those of selected deuterostome and protostome orthologues and the intron-exon structure of the three genes are shown in Figure 1. The homeoboxes of the three genes show clear deuterostome affinities, with closest similarities (as expected) to their echinoid counterparts. The conservation of residues is obvious in the homeodomain, extending to a few residues in both the 3′ and 5′ directions. PmLox and PmCdx possess, upstream of the homeodomain, a hexapeptide with a sequence very similar to other deuterostomian hexapeptides (for example, those of the sea urchin [7]). *PmGsx* lacks this conserved peptide. In both PmGsx and PmLox predicted proteins there is a group of amino acids at the N-terminus that is well-conserved, a fact that has been observed before in other echinoderm homeobox genes [32], but also described in the chordate orthologs (see [33] and Additional file 1: Figure S1).

The case of PmLox is particularly interesting since the hexapeptide is followed by a long stretch of 23 amino acids that seem to be unique to sea star Xlox [see Additional file 1: Figure S1], since we do not find it in the published orthologs of the sea urchin *Strongylocentrotus purpuratus*, the mouse or the mollusk *G. varia*[15]. However, a peptide of similar size is located in the same position in the cephalopod *Sepia officinalis* ortholog (A. Cole; personal communication), suggesting the possibility that the presence of this feature might be an old one, and perhaps has been overlooked in the Xlox genes of other animals. This sea star short peptide is encoded in an extra exon, which is not found in the equivalent position of the sea urchin genome. Since the distance between the hexapeptide and the homeodomain (the linker [34]) seems to be critical for the function of the protein, we suggest that the presence of this long peptide within the linker may have an important impact on its biological function (see also [35]). It is also possible, moreover, that via alternative splicing, the two forms, with and without the extra exon, are both produced in the embryo (a fact that has not been investigated so far).

### *PmGsx*, *PmLox* and *PmCdx* are in cluster in the sea star *Patiria miniata* genome

Sea star ParaHox genes are located in a continuous region of 87 kb (depicted in Figure 1B), without any intervening ORFs in between, as determined by sequencing of clones from a *P. miniata* BAC library. The intergenic distances are 31 kb between *PmGsx* and *PmLox* and 13 kb between *PmLox* and *PmCdx*. As in other deuterostome clusters, the transcriptional orientation of *PmGsx* and *PmLox* are the same while that for *PmCdx* is reversed. As shown in Figure 1B, *PmGsx* is composed of two exons while *PmLox* and *PmCdx* contain three. In the case of the *Cdx* gene, the homeobox is split by one intron (see Figure 1A and B). In *PmLox* and *PmGsx,* the homeobox is located integrally in the last exon (see asterisks in Figure 1B). All exon (protein-coding) and intron sizes are listed in the Additional file 1: Table S1.

Our results demonstrate that *Gsx*, *Xlox* and *Cdx* are clustered in the sea star genome, a situation radically different from what we have seen in the sea urchins [7]. How does this arrangement relate to the situations in other bilaterian taxa? While ParaHox genes are present in all bilaterian phyla, their cluster arrangement has been demonstrated for a limited number of taxa. Intact clusters appear only in some deuterostomes (Hemichordata and Chordata; [5,36]). However, a non-intact ParaHox cluster is clearly present in the annelid *P. dumerilii*[6]. In this latter case, the orthologs of the *Gsx*, *Xlox* and *Cdx* are located within the same chromosome but while the *Pdu-Gsx* and *Pdu-Xlox* genes are neighbors (46 kb apart), the *Pdu-Cdx* gene is located at the opposite end of the chromosome. Interestingly, Hui and collaborators [6] have shown that genes located in the neighborhood of the *Platynereis* ParaHox genes can also be found in close proximity to the ParaHox genes in humans, indicating a unique origin of the ParaHox clusters within the Bilateria (see also [37]). In fact, a thorough analysis of syntenic regions around the ParaHox cluster suggests that the ParaHox cluster was already present in the ancestor of the bilaterians plus the cnidarians [38]. Nonetheless, the ParaHox cluster has been broken repeatedly in different lineages, perhaps due to the activity of transposable elements [39] or other sources of genomic instability (see, for instance [40]). Within the Protostomes we find that, for instance, while *Platynereis* has a (partially) intact cluster, other polychaetes such as *Capitella teleta* have lost any signs of clustering among their ParaHox genes. It is possible that the ancestral condition within the Lophotrochozoa was that of a pair of tightly linked *Gsx* and *Xlox* genes and a dispersed *Cdx*. This is supported by the fact that the genome of the limpet *Lottia gigantea* also shows this ParaHox arrangement (DOE Joint Genome Institute [41]). Within the Ecdysozoa the situation is more simplified, with genes lost in many lineages (*Xlox* in all of them) and the genomic dispersion of *Gsx* and *Cdx* orthologs.

The Deuterostomia, a monophyletic group, comprises four phyla (Hemichordata, Echinodermata, Urochordata and Chordata), with the Xenoacoelomorpha ostensibly as its putative fifth phylum [42]. While, as mentioned, clusters of ParaHox genes have been detected in all these phyla (with the exception of Xenoacelomorpha), there are multiple simplifications occurring in the respective lineages. Intact clusters are seen in vertebrates such as humans, mouse and *Xenopus* (a maximum of one intact cluster remains in each studied species) but in teleost fishes the ParaHox cluster was apparently lost [43]. Interestingly, in the bichir *Polypterus senegalus*, the most basal extant ray-finned fish, the cluster is intact, as it is in the immediate outgroup of the teleost fishes (for instance, the bowfin *Amia calva*; [43]). In all these cases, the gene organization and composition of ParaHox clusters resemble the condition described originally in the amphioxus genome [5]. The transcriptional orientations of the *Gsx* and *Xlox* paralogs are the same, and opposite to that of *Cdx*. A case of disintegration in the cluster is observed in the urochordate *Ciona intestinalis* where the three genes are dispersed in two chromosomes, *Gsx* on chromosome 2q and *Xlox* and *Cdx* on chromosome 14q. *Xlox* and *Cdx* are separated, however, by 240 kb with many intervening genes in between, a sign of progressive dispersion [44]. Hemichordates have kept their cluster intact, at least in the Ptychoderidae (reported in [36,45]).

### Temporal expression pattern of the three ParaHox genes during sea star embryonic development

**Figure 2:**
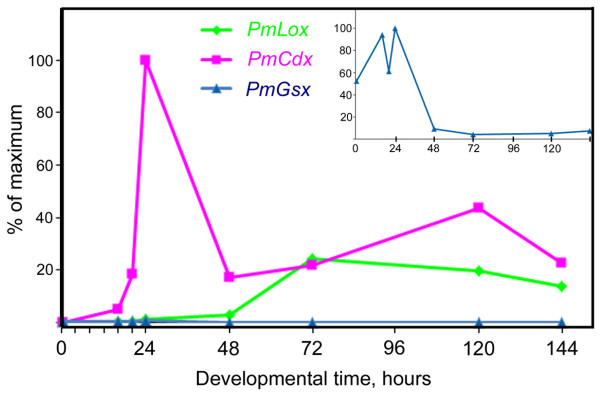
**Relative temporal expression profiles of ParaHox genes during *Patiria miniata *development.** The graph shows the relative transcript abundance normalized against ubiquitin mRNA levels during sea star development. mRNA levels were measured by QPCR from cDNA templates prepared from whole embryos at the indicated developmental times. The results are expressed as percentage of the maximum value corresponding to *PmCdx* level of expression at 24 hpf. Due to the low levels of *PmGsx* transcript abundance, a separate graph has been created for *PmGsx* alone (see inset) expressing the results as percentage of *PmGsx* maximum level of expression (24 hpf). *PmLox* and *PmGsx* curves have been obtained repeating the experiments with two independent sets of primers. For the detailed experimental procedure see the relative section in Methods. Hpf, hours post fertilization.

In order to examine the dynamics of *P. miniata* ParaHox gene expression during embryonic development, we analyzed the temporal expression profiles using quantitative PCR, as described in Methods. One striking finding is the *PmGsx* maternal expression, something that has never been reported in any other taxon. *Gsx* transcript levels do not change significantly during the first 24 hours, with some fluctuations imposed by the procedural detection limit (Figure 2). Given the constancy of this level it is very possible that we are just detecting the maternal message over the whole period (without any zygotic contribution). After 24 hours post fertilization (hpf) (see the small insert in Figure 2) *PmGsx* expression levels drop progressively over the next day until it is undetectable for the subsequent developmental stages. A possible activation of *Gsx* transcription in later larval stages or in the adult sea star cannot be excluded. *PmLox* expression is not maternal and starts from 24 hpf when a slow accumulation of messages is detectable. From 48 hpf, *PmLox* levels of expression strongly increase reaching a maximum at 72 hpf. After this stage and until the last time point analyzed (six day larva) transcript levels decrease continuously but some expression of the gene is still detectable. The *PmCdx* mRNA is not present in eggs. After the first 20 hours of embryonic development the levels of *PmCdx* transcripts increase progressively reaching maximum accumulation at 24 hpf. After this time the levels start to drop, and they do so for the next 24 hours, until 48 hpf. During the next three days, until day five post fertilization, a second wave of expression of *PmCdx* is detectable. The expression levels increase again reaching a maximum (at five days post fertilization (dpf)) that is less than half of what was detected at 24 hpf. Subsequently, up until day six, the levels of *PmCdx* mRNA seem to decrease although the transcription of the gene is still ongoing.

Clearly, during the time frame of these experiments, which spans embryogenesis in its entirety, *PmLox* has a single peak of maximum accumulation at day three, while *PmCdx* seems to accumulate in two different waves, with peaks of accumulation at days one and five, respectively. The levels of *PmGsx* mRNA are always low, due most probably to solely maternal contribution. Based on the QPCR data, and understanding that *PmGsx* is only maternal, although detectable until 24 hpf, we would suggest that *PmCdx* is the first ParaHox gene to be activated during sea star development, followed by *PmLox*, which is turned on more than one day later. Moreover, a possible role for *PmGsx* in advanced larval stages or in adult body formation would indicate that *Gsx* is the last ParaHox gene to be activated. Our data show that the sea star ParaHox transcription factor temporal activation resembles the sequence of activation observed in chordates [11] while it is inverted for the sea urchin orthologs [7]. Moreover, the sea star ParaHox cluster characteristics support the theory that mechanisms producing temporal colinearity are likely the major constraining forces on gene cluster maintenance [46-48]. In the sea star, both the temporal order of expression (at least for *Xlox* and *Cdx*) and the cluster organization, typical of Chordata, seem to be conserved, while in the sea urchin, the temporal colinearity (assuming the cluster arrangement in chordates) is inverted and the cluster is broken.

### The spatial expression domains of the ParaHox genes during sea star embryonic development

**Figure 3:**
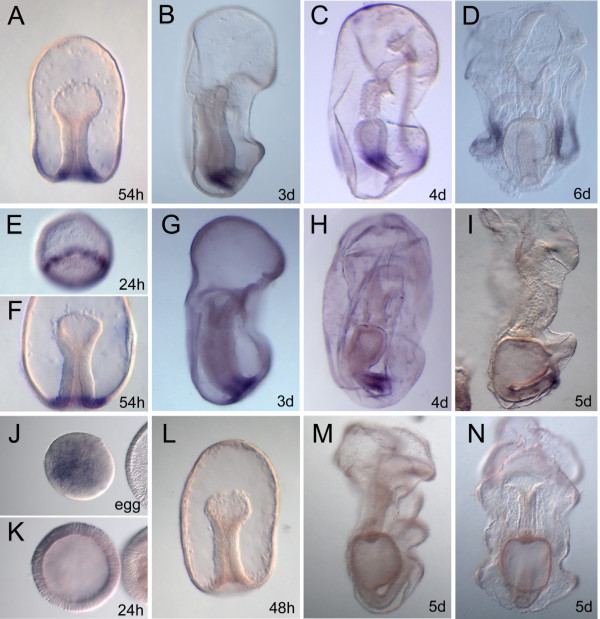
**Spatial expression patterns of ParaHox genes during *Patiria miniata *development.** Panels **A**, **B**, **C** and **D** show the expression of *PmLox* gene. Panels **E**, **F**, **G**, **H**, **I** correspond to *PmCdx* expression. Panels **J, K, L, M, N** provide the expression domains for *PmGsx*. In panels **A**, **D**, **F**, **L**, **N** and **J**, embryos and larvae are in frontal view; in **B**, **C**, **G**, **H**, **I**vand **M**, embryos and larvae are in lateral view; **E** and **K** are vegetal views of blastula embryos. Developmental stages are indicated in each panel.

*PmGsx*, *PmLox* and *PmCdx* spatial expression patterns have been analyzed by whole mount *in situ* hybridization experiments at different stages of development, from egg until six-day larva, and the results are shown in Figure 3. *PmLox* transcripts are detectable in sea star embryos only after 48 hpf and the first expression domain is localized in the ectoderm of the mid-gastrula stage embryo. The detailed description of *PmLox* ectodermal expression during *P. miniata* development is provided below. From 52 hpf *PmLox* starts to be expressed in a group of cells localized in the posterior region of the archenteron (Figure 3A). In the 60-hour embryos, the transcript levels increase in both the ectodermal and endodermal domains of expression and at 72 hpf, when the archenteron is completely invaginated, *Xlox* expression is confined to the midgut-hindgut boundary region (Figure 3B) where it is detectable also in the completely differentiated 4-day larval gut (Figure 3C). In the 5- and 6-day larvae the gene is strongly expressed in the ectodermal domain (Figure 3D) and a very weak signal is visible in cells localized between the stomach and intestine, a region where a pyloric sphincter is usually present in other animals. *PmCdx* expression is first detected in a ring of cells localized in the vegetal half of the 24-hour embryo and surrounding the blastopore (Figure 3E). Following the description of vital staining results performed in another sea star species, *Asterina pectinifera*[49], the cells expressing *PmCdx* likely represent veg1 descendants. *PmCdx* expression persists in the vegetal side of the embryo and it is restricted to the blastopore region in the mid-gastrula embryos (Figure 3F). At late gastrula stage, when the archenteron is completely invaginated, *PmCdx* expression is detectable in the portion of the gut that will give rise to the hindgut and in the anus (Figure 3G). In the 4-day larva, when the gut is completely differentiated, the signal is clearly detected in the intestine and it extends until the anus (Figure 3H). In the 5- and 6-day larvae, the expression is reduced in the intestine and remains strong in the anal cells (Figure 3I). The expression of *PmGsx* is visible in the egg (Figure 3J) as maternal message, without any regional localization. Later on, the levels remain low, barely above the minimum levels needed for *in situ* detection until 24 hpf (Figure 3K). After 24 hpf no expression is detectable through *in situ* hybridization at any of the examined stages (up to the 5-day larva, Figure 3L-N), thus corroborating the temporal expression profile obtained by QPCR (Figure 2).

Considering all our results together, some very interesting features can be highlighted. First of all, conservation in the expression domains of the sea urchin and sea star orthologs is of note. Further, the expression of the sea star *Cdx* in the blastula stage, well before gastrulation has been initiated, implicates a function for *Cdx* that is not present in sea urchins. However, an early activation of *Cdx* in development, prior to its recruitment in posterior gut patterning, appears as a common feature among chordates: in murine embryos the first embryonic territory of expression for *Cdx* genes is the posterior primitive streak [50]; moreover, in *Xenopus tropicalis* the three orthologs *Cad1*, *Cad2* and *Cad3* are first expressed in the early gastrula around the blastopore and later in the posterior embryo, including the gut [51]; finally, the first expression of the amphioxus *Cdx* orthologue is detected in a ring of cells surrounding the blastopore opening [11]. The conservation of *Cdx* gene expression around the primitive streak of the early mouse and the blastopore of frog, amphioxus and sea star embryos, suggests the existence of an ancestral role for *Cdx* in the early stages of deuterostome development. This function has been lost in the sea urchin embryo where only a late role for *Cdx* in patterning posterior structures, in this case the gut, has been found [7].

### *PmLox* and *PmCdx* dynamic relative expression along the sea star developing gut

**Figure 4:**
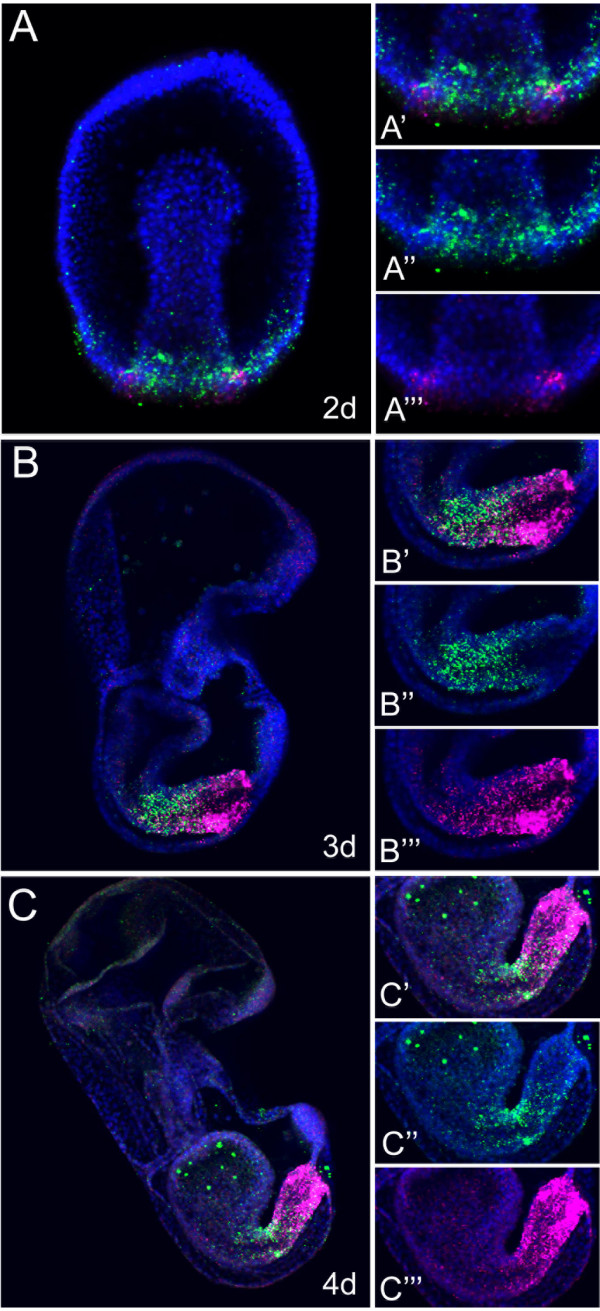
***PmLox *and *PmCdx *relative expression domains in the sea star developing gut. (A, B, C)** Double *in situ* hybridization of *PmLox* (green) and *PmCdx* (magenta) expression domains coupled with nuclei staining (blue, obtained with DAPI) in sea star embryo and larvae. In A, a gastrula embryo is shown in frontal view. In B and C, larvae are shown in lateral view. Developmental stages are provided in each panel. On the right of each developmental stage, a magnification of the gut domain expressing the two genes is provided, showing first both channels **(A’, B’, C’)**, then the green channel only **(A”, B”, C”)** and then the magenta panel **(A”’, B”’, C”’)**; nuclear staining is shown in blue. All the pictures represent full projection of confocal z-series. d, days; DAPI, 4',6-diamidino-2-phenylindole.

In Figure 4 a double fluorescent *in situ* hybridization experiment performed on 2- to 3- and 4-day sea star embryos is shown. *PmLox* (in magenta) and *PmCdx* (in green) occupy distinct territories of expression in the gastrula embryo: both genes are expressed in the posterior portion of the archenteron with *PmCdx* positive cells positioned toward the posterior side of the gut (Figure 4A) and *PmLox* expressing cells adjacent to *PmCdx* but localized more anteriorly. In the 3-day larva, *PmLox* and *PmCdx* extend their domains of expression with *PmCdx* covering most of the intestine, until the blastopore, and *PmLox* being localized in the anterior part of the hindgut (Figure 4B). In the 4-day bipinnaria larva, when a large distinct stomach is visible, *PmCdx* transcripts occupy most of the intestine, showing a posterior to anterior gradient of abundance (Figure 4B); the gradient decreases along the intestinal tube reaching the minimal levels in the cells positioned at the boundary between stomach and intestine, where both *PmCdx* and *PmLox* genes are expressed (see Figure 4B). A gradient of expression for *PmCdx* along the embryonic antero-posterior (A-P) axis has been described in several animals both at the mRNAs level [52-54] and at the protein level [52,55]. In this context, Cdx proteins are considered as possible morphogens whose gradient is responsible for a proper distribution of target gene transcription along the A-P axis of the embryo; this role is probably conserved for the sea star ortholog PmCdx. Moreover, the partial overlapping of *Cdx* and *Xlox* transcripts in the intestine of the sea star larva opens a number of hypotheses about the possible regulatory interactions between the two ParaHox genes. In sea urchins, a necessary role for *SpLox* in the activation of *SpCdx* transcription and for *SpCdx* in the repression of *SpLox* transcription within hindgut cells has been demonstrated [8]. We cannot exclude the existence in the sea star embryo of a similar function for *PmLox* on *PmCdx*, although, since no co-expression with *PmCdx* is detected at the onset of *PmLox* expression in the posterior gut we would have to invoke a signaling event mediating such regulation. In fact, *PmCdx* dynamic of transcript accumulation consists of two waves of activation, the second happening after *PmLox* onset of transcription. On the other hand, a repressive function for *PmCdx* on *PmLox* transcription in the posterior larval intestine is likely occurring in the sea star, as it happens in the sea urchin and apparently in the mouse: a similar dynamic has been described in *Cdx2* mouse null mutants where ectopic expression of *Pdx1* was found in the intestine [56].

### *PmLox* ectodermal domain of expression

*Xlox* expression in neural territories has been reported in many animals, protostomes and deuterostomes alike [6,11,18,20,57]. Within the echinoderms it has been shown that in the sea urchin *Strongylocentrotus purpuratus SpLox* is specifically expressed in cells localized below the ciliary band, in a position corresponding to the lateral ganglion [19].

**Figure 5:**
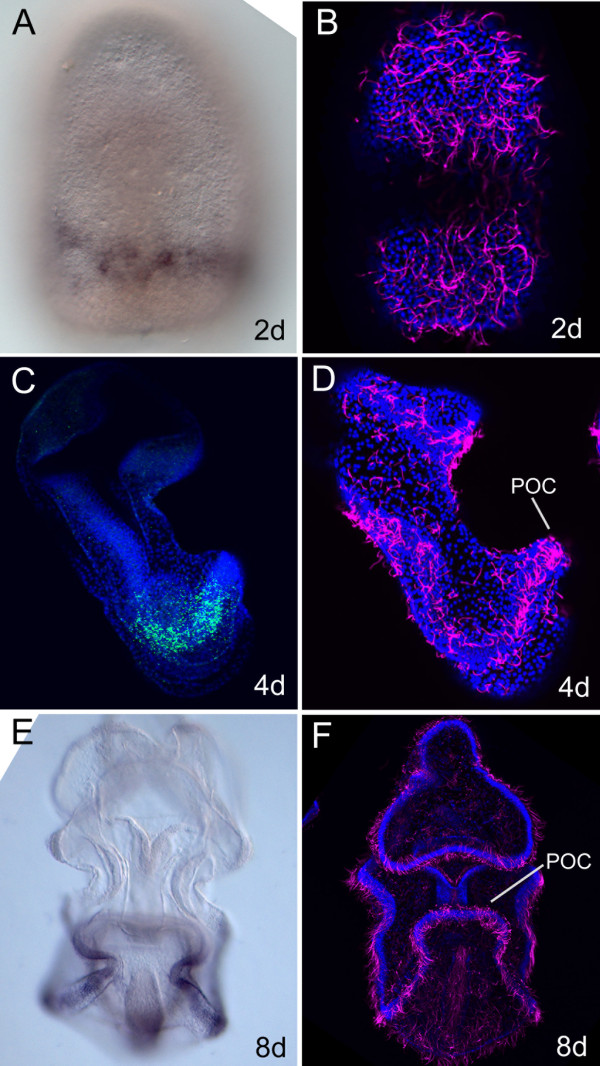
***PmLox *ectodermal domain of expression. (A**, **C**, **E)***PmLox in situ* hybridization developed with alkaline phosphatase methodology **(A, E)** and with fluorescent tyramide cy5 methodology coupled with DAPI staining (**C**, *PmLox* transcripts in green, nuclei in blue); **B**, **D** and F show acetylated tubulin immunostaining (in magenta) coupled with nuclear staining (in blue). **A**, **B**, **E**, **F**vembryos and larvae are shown in frontal view; **C** and **D** larvae are shown in lateral view. Developmental stages are indicated in each panel. d, days; DAPI, 4',6-diamidino-2-phenylindole.

In this study we demonstrate that *Xlox* ectodermal recruitment in development is also conserved in the asteroid *P. miniata*. In Figure 5, a detailed analysis of *PmLox* transcript localization in the ectoderm of the sea star embryo and larva (A, C, E) is shown, with respect to the position of the developing ciliary band (detected by acetylated tubulin immunostaining in B, D, F). The *in situ* hybridization experiments revealed that the first expression for *PmLox* is detected in a group of ectodermal cells in the 2-day gastrula embryo. *PmLox* positive cells are localized in the oral ectoderm of the embryo, arranged in a semicircle located at the level of one third of the invaginated archenteron (considering the blastopore as reference point) (Figure 5A). Acetylated tubulin immunostaining in the 2-day embryo clearly shows the absence of a distinctive ciliary band (Figure 5B), as previously reported for the sea star *A. pectinifera*[58]. Our results show that although at the cell type level no specialization is detectable in the ectodermal cells of the 2-day gastrula embryo, a differential regulatory state is already present in a subpopulation of ectodermal cells expressing exclusively *PmLox*. In fact, the existence of subdomains in the ectoderm of the pre-larval sea star embryo has already been described by Yankura and collaborators [59] where a number of exclusive gene expression domains have been identified in sub-regions of the ectoderm. A few hours later, at 54 hpf, *PmLox* positive cells lose the aligned organization, probably migrating in opposite directions, and finally reaching separate locations in the ectoderm that will contribute to the formation of the postoral ciliary band (POC), which subdivides the ectodermal epithelium in oral and aboral epidermis. In the 4-day bipinnaria larva, *PmLox* ectodermal expression is localized in a relatively large number of cells of the POC in each of the two symmetric sides of the larva (Figure 5C, showing the left side of the larva); the ectodermal cells expressing *PmLox* are localized in a restricted region of the POC, localized at the level of the vegetal folds (Figure 5D). In the 8-day brachiolaria larva, the *PmLox* ectodermal domain extends to a larger number of cells distributed along the vegetal portion of the POC, following the ectodermal lateral loops that will give rise to the future appendages; no *PmLox* transcripts have been detected in the frontal fold of the POC (Figure 5E). The *PmLox* domain of expression in the 8-day brachiolaria larva clearly corresponds to a sub-domain of the POC (Figure 5E and F), suggesting a potential role of the PmLox transcription factor in the development of the sea star larval nervous system and possibly reflecting an ancient function for this gene in the specification of neural cell types.

### Evolutionary implications: changing the genomic organization and the activities of ParaHox genes within the Echinodermata

Over the last few years it has been shown that ParaHox genes are arranged in genomic clusters in several bilaterian taxa, a fact that might have important consequences for their regulation. However, this sometimes tight arrangement is broken in many lineages. One clear example of this evolutionary phenomenon is shown in the echinoderms. We have shown in the past that the three ParaHox genes are not clustered in the sea urchin genome [7]. Here, instead, we demonstrate that a member of the asteroids (*P. miniata*) has kept the ancestral condition, the presence of a tight cluster of genes [37], a condition retained in the sister group of all echinoderms, the hemichordates [45]. The evolutionary history of these genes and their putative ancestral roles merit some further comments, which are given in the next paragraphs.

When evaluating the dynamics of some genomic arrangements, we think it is interesting to note that the partial break of ParaHox clusters, or their eventual disintegration, seems to occur in parallel, in the various taxa where these events take place, to the break and dispersion of its sister cluster, the HOX. The modification of the cluster seems to be related to the average substitution rate of the different taxa, as seen in some metazoan phylograms [60], such that faster evolving clades are more prone to cluster modification. An example in this context would be the sea urchins (echinoids) where the Hox cluster has been reorganized through breaks and the loss of genes (Hox4) or the fusion of transcriptional units (Hox5). In parallel, a dispersion of the three ParaHox genes within the genome has occurred. Interestingly, one break point in the echinoid Hox cluster occurs at the Hox4 locus, with the consequence that this gene is lost [61]. In sea stars, Hox 4 is retained [62] suggesting that, perhaps, this break is not present in asteroids. If this were the case, and taking into account what we now observe in the *Patiria* ParaHox, we would be facing another case of parallel Hox/ParaHox clusters evolutionary histories. Hox and ParaHox clusters would be retained in asteroids but broken (to different degrees) in echinoids.

**Figure 6:**
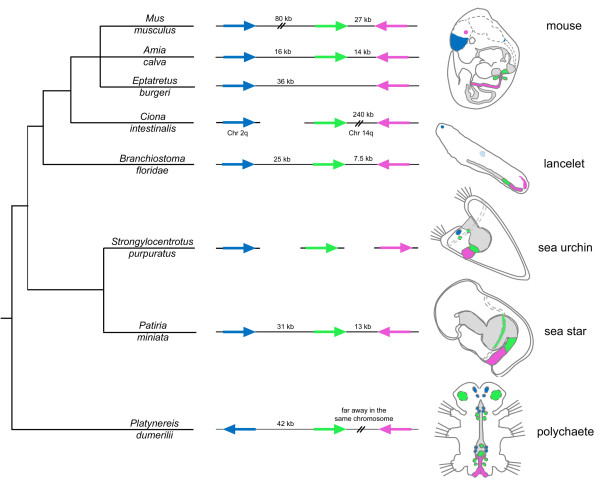
**Evolution of the ParaHox gene cluster and relative expression domains in bilateral animals.** Schematic representation of ParaHox genomic organization and expression patterns in several bilateral animals: the mouse *Mus musculus* [9,17,23,33,63], the bowfin fish *Amia calva* [43], the hagfish *Eptatretus burgeri* [64], the lancelet *Branchiostoma floridae* [5,11], the sea urchin *Strongylocentrotus purpuratus* [7,19], the sea star *Patiria miniata* (present study) and the polychaete *Platynereis dumerilii* [6]. Arrows indicate ParaHox genes and their orientation in the genome; a continuous line below arrows indicates an intact cluster. The cartoons on the right side of the panel schematize the domains of expression of ParaHox genes in representatives of some of the bilaterian groups. Mouse, amphioxus, sea urchin and sea star embryos are in lateral view; the polychaete embryo is in frontal view. Nervous system domains are depicted with dashed lines. Endodermal structures are in gray. Gsx expression is shown in blue, Xlox in green and Cdx in magenta. Amphioxus Gsx expression in the hindbrain is depicted in light blue, as at the stage of development represented here, this domain of expression is fading out [11]. kb, kilobases; Chr, chromosome. Double-parallel lines indicate long genomic distance.

An essential issue in our current understanding of the role that clustering has in the expression of genes is whether there is a correlation between gene positions within the cluster and their respective spatial domains of expression. This has been amply debated within the context of Hox gene activities and the evolution of the HOX cluster, but it is equally relevant to the workings of the smaller bilaterian ParaHox clusters. We have investigated here the relationships between ParaHox gene activities and the presence of a cluster arrangement. It is remarkable that what we observe in asteroids, with respect to the gene order and activities, is notably different from what has been observed in the echinoids, a fact that needs a clear analysis. It has been demonstrated that *Xlox* and *Cdx* genes in the sea urchin genome are localized on two different scaffolds of at least 300 kilobases [7] but a correlation between the temporal and the spatial sequence of activation is still observed: *SpLox* is expressed earlier during development and in a domain of expression anterior to *SpCdx* that is activated (requiring *SpLox* regulatory input) a few hours later. In the sea star we found a similar relative transcript distribution of *PmLox* and *PmCdx* along the antero-posterior axis of the larval gut, with *Cdx* expressed in a more posterior domain than *Xlox* (Figure 4). What is different is the temporal sequence of activation of the two genes, in the sea star resembling the chordate typical order while in the sea urchin the pattern is inverted. As mentioned above, the analysis of the BAC clones isolated from a *P. minata* genomic library shows that all three ParaHox genes are clustered in the sea star genome. The relative domains of expression in the two echinoderm classes, as well as in other bilaterian phyla, are schematically shown in Figure 6. This scheme includes all the phyla for which complete information is available. The comparison of genome organization and expression patterns of all ParaHox genes included in Figure 6 is allowing us to engage in some speculative considerations.

Given the presence of an intact cluster in deuterostomes and of a partially intact cluster in protostomes, we can infer that the common ancestor of all Bilateria had a ParaHox cluster composed of three genes, *Gsx*, *Xlox* and Cdx. This fact is supported by the shared presence of similar genes in the genomic neighborhood of *Platynereis* and human ParaHox genes [6]. The cluster itself might have originated from a single precursor gene that was duplicated in *cis*, or, alternatively, it was derived, as a whole, from the duplication of a ProtoHox ancestral cluster [65]. This tight genome arrangement was kept in several lineages although in others, perhaps as a result of a relaxed selection, it was broken into pieces. Within the echinoderms, we have a clear example of the dynamics of the cluster in evolutionary time. We show that asteroids (sea stars) maintain a tight chromosomal arrangement of the three genes while in the echinoids (sea urchins) the genes are dispersed in the genome (this paper and [7]). Interestingly, and independently of the genomic arrangements, there are some aspects of ParaHox gene regulation that are kept, even in the absence of an intact cluster. The most important of these aspects is the nested nature of their endodermal expression domains. When it comes to spatial domains within the gut, in almost all cases studied the expression patterns of the genes (but fundamentally *Xlox* and *Cdx*) are maintained, with *Xlox* being expressed anteriorly to *Cdx*. The presence of *Gsx* in the bilaterian guts is less conserved. It is very possible, as it has been shown for the Hox genes, that a tight cluster arrangement is a prerequisite for a strict temporal control of the genes expression, while the relative spatial domains can be preserved in the absence of tight clustering. This suggested hypothesis has not been tested for the ParaHox genes, since most of the observations are purely circumstantial at this point. No clear kinetic studies allow us to confirm or reject such an assumption at the moment.

Given the use of ParaHox genes in gut patterning, and their historical birth in the *cis*-duplication of a precursor gene, it is reasonable to assume a scenario in which the bilaterian ancestor used a single proto-parahox gene (one possible candidate being the Placozoa Trox-2 gene [66]) to control the specification of the gut tissue (although not excluding its use in other germ layers). This precursor gene was duplicated in cis, giving rise to the three extant ParaHox genes, *Gsx*, *Xlox* and *Cdx*, linked in the genome. These genes evolved their functions by sub-functionalization [67] and, perhaps in parallel, giving rise to different functional domains within the gut. In some cases the domains were maintained by cross-regulatory interactions (sea urchins, for instance), although this could also be maintained even in the event of cluster disintegration. The cluster in itself proved quite flexible, in terms of size, as can be seen clearly in Figure 6. Strikingly, the changes in gene spacing within the ParaHox cluster occurred without the incorporation of new ORFs inside it. However, the few analyses we have on the occurrence and nature of the intervening sequences have demonstrated the presence of many repetitive elements, or rests of transposable elements in these regions, suggesting that one mechanism controlling the cluster size might be through the expansion of repetitive sequences or the inclusion (transposition) of new mobile elements [39]. At the same time these elements could provide the structural basis for the later cluster disintegration in specific lineages (as seen in the Hox cluster; [7]).

We should emphasize, once more, that it is becoming clear, from the analysis of different animal genomes, that there is a parallel disintegration of Hox and ParaHox clusters in some lineages. This might not be so surprising being most probably the reflection of the evolutionary rates of change in the DNAs of each lineage (seen, for instance, as branch lengths in phylogenetic analysis). The cases of tunicates, or acoels, are paradigmatic, but it is a fact also demonstrated here for the echinoderms, where a disintegration of the ParaHox cluster has accompanied the parallel rupture of the Hox clusters. This leads us to the inescapable conclusion (not surprising, though) that the dynamics of cluster evolution are a consequence of the global dynamics followed by the genome over evolutionary time.

## Conclusions

Recent advances in genome sequencing technologies have produced a flurry of genomic data from many, previously inaccessible, animal systems. This has generated a renewed interest in the dynamics of genome evolution and in the relationship between changes in the genome and different morphological transformations.

Among those features that are now being thoroughly investigated, the relationship between gene clustering and regulation is of special relevance. Hox and ParaHox genes have been classical examples of vectorial patterning systems with complex regulation. However, most of the data supporting current models of Hox and ParaHox function have been obtained in a relatively small group of animals, mainly insects and chordates. It is clear that a full understanding of how these gene families (and their functions) have evolved over time will be possible only when a wider selection of animal models is considered.

Here, we present a thorough investigation of one such group of genes, the ParaHox. We have cloned and thoroughly characterized the ParaHox group of genes in the sea star *P. miniata*. Moreover, we have studied their genomic organization and the expression domains of each gene, in space and time, during embryogenesis. We show that sea stars organize the ParaHox genes in a single, compact cluster, reflecting their basal position within the Echinodermata and highlighting the more derived nature of the related group of echinoids. We show that both gene cluster disruption and the changing patterns of gene expression are happening within one single phylum, a clear sign of the dynamic nature of genomes during evolution.

## Methods

### Cloning of *PmGsx*, *PmLox* and *PmCdx*

Universal degenerate primers amplifying the ANTP family homeoboxes were used on cDNA synthesized from a mixture of 3- and 4-day old *P. miniata* embryo RNA, following the protocol published in Martinez *et al*. [32]. The PCR product mix was cloned into the pGEM-T Easy vector (Promega, Madison, WI, USA) and a large number of colonies were screened leading to the isolation of the clones containing *PmLox* and *PmCdx* homeoboxes. Using as probe the *PmLox* homeobox, a clone containing a 1,454 bp *PmLox* fragment was successfully isolated from a *P. miniata* (3-day embryo) cDNA library. A 3′-RACE strategy was followed to obtain a longer portion of *PmCdx*. In particular, a mix of 3- and 4-day RNA was used as template for the 3′-RACE performed using the kit 3′-RACE System for Rapid Amplification of cDNA Ends (Invitrogen, Carlsberg, CA, USA); two primers, a first forward primer and a second nested primer were designed based on the available sequence (PmCdx3′-RACE: ACATCACCATCAGACGCAAG, PmCdx3′n-RACE: GGGACTATCCGAGAGACAGG) and the PCRs were conducted following the manufacturer’s instructions. The amplified product was cloned into Topo-TA cloning vector (Invitrogen). A fragment of 1,221 bp of *PmGsx* was cloned from *P. miniata* genomic DNA designing specific primers on the sequence of the BAC containing the three *P. miniata* ParaHox genes (PmGsxF1: AAAACACCGAAAATTGCAAAG, PmGsxR1: AGTTTTGCGGCCACTTTCTA).

### cDNA and BAC filter screenings

*P. miniata* cDNA library arrayed filters were screened using as probes *PmLox* and *PmCdx* homeoboxes, while the BAC library filters were screened using the *PmLox* cDNA clone and *PmCdx* fragment obtained by 3′-RACE. The screenings were performed following the protocol described in Martinez *et al*. [68].

### Sea star embryos, *in situ* hybridization and immunostaining experiments

Adult *P. miniata* were obtained from Patrick Leahy (Kerchoff Marine Laboratory, California Institute of Technology, Pasadena, CA, USA) and housed in circulating sea water aquaria in the Stazione Zoologica Anton Dohrn of Naples. Gametes were obtained following the procedure described in Hinman *et al*. [69] and embryos were raised at 15°C in filtered sea water diluted 9:1 with de-ionized water. After day 4, larvae were fed daily with a mix of *Isochrysis galbana* and *Rhodomonas lens* algae. For *in situ* hybridization, probes were transcribed from purified PCR amplified template DNA, using digoxygenin-11-UTP (Roche, Indianapolis, IN, USA) or labeled with dinitrophenol (DNP) (Mirus, Madison, WI, USA) following kit instructions. Whole mount *in situ* hybridization experiments were performed as described in Hinman *et al*. [69], with the modifications suggested in Yankura *et al*. [59] for the double fluorescent *in situ* hybridization procedure. For the acetylated tubulin immunostaining, embryos and larvae were fixed overnight at 4°C in PBS with 2% paraformaldehyde (PFA), washed in phosphate buffered saline with Tween (PBST) several times, incubated in the blocking solution (5% sheep serum, 1X PBST) for one hour at room temperature and then incubated overnight in the blocking solution with a dilution 1:250 of the mouse monoclonal anti acetylated tubulin antibody (Sigma-Aldrich, St Louis, MO, USA). The next day the embryos were rinsed several times with PBST, incubated in the secondary antibody, the AlexaFluor 488 goat anti- mouse immunoglobulin G (IgG) (Molecular Probes, Eugene, OR, USA), and after one hour washed to remove the unbound antibody. For the fluorescent *in situ* hybridization and the immunostaining, embryos were examined and images obtained with a Zeiss confocal laser-scanning microscope LSM 510. For the colorimetric *in situ* hybridization, embryos were observed and pictures made with the use of a Zeiss digital camera (Axiocam) mounted on a Zeiss Axioimage 2 MOT microscope operating in DIC mode.

### Quantitative polymerase chain reaction

Total RNA was collected from 300 embryos for each analyzed stage with the use of the Ambion^®^ RNAqueous-Micro Kit (Life Technologies, Carlsberg, CA, USA) and cDNA synthesized with the SuperScript^®^ VILO™ cDNA Synthesis Kit (Invitrogen) following the manufacturer’s instructions. Temporal accumulation of messages was monitored using real-time quantitative PCR (QPCR). Specific primer sets for each gene were designed on separate exons (for *PmLox* and *PmGsx* two sets of primers were designed and used in the QPCR experiments). Reactions were performed using the ViiA 7 REAL TIME PCR with SYBR Green chemistry (Applied Biosystems, Foster City, CA, USA). Ubiquitin was used to normalize gene expression data following Hinman *et al*. [69]. Sequence of the primers used for QPCR: PmLoxQF1: GCCGCATCATCATCATACAC; PmLoxQR1: ACATATGAGCGTGCGATTTG; PmLoxQF2: CACGCTCATATGTGGAAAGC; PmLoxQR2: CGCTTGTTCTCGTCAAAGTCT; PmCdxQF: ACCGGAGATGGTCCTGAAC; PmCdxQR: GCATGAAGACAGGGCAGTTT; PmGsxQF1: AGACCCGAGGAGACTCCAAT; PmGsxQR1: ACATGTTGGAGGCGAACTCT; PmGsxQF2: CGGCGGGTCAAGTACAAG; PmGsxQR2: CAATCTCTCTCCGGCTGC).

### BAC sequencing and assembling

A mixture of the BAC clones, CeCl-purified, positive for both *PmLox* and *PmCdx*, was sequenced at the European Molecular Biology Laboratory (EMBL) Genomics Core Facility using a massive parallel sequencing platform (Illumina, San Diego, CA, USA). Sequence gaps were filled using specific primers and targeted capillary electrophoresis (Abi3730DNA analyzer, Life Technologies, Carlsberg, CA, USA) at the Molecular Biology Service of the Stazione Zoologica Anton Dohrn of Naples. The assembly of the individual reads was performed by the EMBL bioinformatics service, and further assembly including the sequences obtained through the walking strategy was performed using the *SeqMan* Pro Lasergene suite program. A prediction of the exon-intron junctions was performed using Genescan software and validated with the available cDNA sequences. The sequences of the complete coding region of the three genes have been deposited in Genbank [KC551919, KC551920, KC551921] and are reported in Additional file 1.

## Abbreviations

ANTP: Antennapedia; A-P: Antero-posterior; BAC: Bacterial artificial chromosome; bp: Base pair; CNS: Central nervous system; DAPI: 4',6-diamidino-2-phenylindole; DNP: Dinitrophenol; dpf: Days post fertilization; hpf: Hours post fertilization; Mya: Million years ago; ORF: Open reading frame; PBST: Phosphate buffered saline with Tween-20; PCR: Polymerase chain reaction; POC: Post oral ciliary band; UTR: Untranslated region; 3' RACE: Rapid amplification of cDNA ends.

## Competing interests

The authors declare that they have no competing interests.

## Authors’ contributions

MIA and PM conceived and supervised the project. RA performed all the experimental work. The authors contributed equally to the interpretation of the results and the writing of the manuscript. All authors read and approved the final manuscript.

## Supplementary Material

Additional file 1: Table S1 Exon-intron sizes of *Patiria miniata* ParaHox genes. **Figure S1.** Sequences of *P. miniata* ParaHox genes. **Figure S2.** Phylogenetic analysis of *P. miniata* ParaHox genes together with all ANTP genes from the sea urchin *Strongylocentrotus purpuratus* (Sp), the protostome *Drosophila melanogaster* and the cnidarian *Nematostella vectensis* (Nv), rooted on *Lim*. For methods, data set and abbreviations, see Arnone MI, Rizzo F, Annunziata R, Cameron RA, Peterson KJ, Martinez P. Genetic organization and embryonic expression of the ParaHox genes in the sea urchin *S. purpuratus*: Insights into the relationship between clustering and colinearity. *Dev. Biol.* (2006) 300, 63–73. Click here for file
