# Supplementary material for: Intact cluster and chordate-like expression of ParaHox genes in a sea star
Source: BMC Biol. 2013 Jun 27;11:68. doi: 10.1186/1741-7007-11-68 (PMC3710244; doi:10.1186/1741-7007-11-68)
Supplement: Additional file 1: Table S1 — Exon-intron sizes of Patiria miniata ParaHox genes. Figure S1. Sequences of P. miniata ParaHox genes. Figure S2. Phylogenetic analysis of P. miniata ParaHox genes together with all ANTP genes from the sea urchin Strongylocentrotus purpuratus (Sp), the protostome Drosophila melanogaster and the cnidarian Nematostella vectensis (Nv), rooted on Lim. For methods, data set and abbreviations, see Arnone MI, Rizzo F, Annunziata R, Cameron RA, Peterson KJ, Martinez P. Genetic organization and embryonic expression of the ParaHox genes in the sea urchin S. purpuratus: Insights into the relationship between clustering and colinearity. Dev. Biol. (2006) 300, 63–73. [file 1741-7007-11-68-S1.pdf]

# Annunziata et al, Supplementary material

## Table1. Exon-intron sizes of *Patiria miniata* ParaHox genes

| Gene | exons | introns |
|---|---|---|
| **PmCdx** | exon1: 435 bp<br>exon2: 146 bp<br>exon3: 412 bp | intron1: 4653 bp<br>intron2: 1139 bp |
| **PmLox** | exon1: 587 bp<br>exon2: 69 bp<br>exon3: 463 bp | intron1: 25264 bp<br>intron2: 6262 bp |
| **PmGsx** | exon1: 475 bp<br>exon2: 368 bp | intron1: 2210 bp |

Data are based on comparison of cDNA and genomic sequences. 3′ and 5′ UTRs are not included.

## **Fig. 1**. **Sequences of *P. miniata* ParaHox genes**

## PmCdx

**>*PmCdx* exon1**
ATGTACCGGCACCCGAATCCAGCGGCACAGGCCTACTCCTCTTTCAACTACAGCCAGACTCCGACCAGCCAGTACCCGTCCGAGTATTC
CCCGTCAGCAACCGGCTCGTATCAGGTCTCGAGCAGCCTGGATGCCCAGCAGCAACAGTCGGTCCCGACCACCAACTCTTCCTGGGCCT
CGGCGGGCTTCCCAACCTCCGTGGACTGGGCCACGGCGTCCCAAGACCCGTTCGGTGCCGGCACCTTCCCCGGCCAAACACCGGCCGGA
CCTATGAGCAGTTCACCGTACAACTCCGAGGATCTGGACTACTACCAGGGCTTCGGCCAGCAGACCGCCGTCAATAGCGTCTCTCAGGC
CCTCCACCGGCCGGCGATGCGCTCGACTTACCACGATTGGATGAGGTCCACTTCGTACCGGGTCAACCCTCAAACAGGT
**>PmCdx exon1**
MYRHPNPAAQAYSSFNYSQTPTSQYPSEYSPSATGSYQVSSSLDAQQQQSVPTTNSSWASAGFPTSVDWATASQDPFGAGTFPGQTPAG
PMSSSPYNSEDLDYYQGFGQQTAVNSVSQALHRPAMRSTYHDWMRSTSYRVNPQTG
**>*PmCdx* intron1**
TCGTATACAAAGTTGTGAATTCTTAAAATGACGAAATTAGGTACTAAATTATATTCGTTTACAAATTTATGTAAGTAATTTTTTCAAAG
TTCATTATCTCCTTCTGATAACTTATCTGATATAAGACAATCAATTTATCATTAGTTCATTTTTTGTTCTGTCTAGGCTGTTCGAATGG
ACTTTTTTTTTTAAATGACTCTGTTTTTAAGTAAGAAAAGTAACACATTTATCAGCTTATTGTTTAGTCCAGCATATGACTCTGGTTTT
TATTTTTATGTTGCATTTTTATTTTCAAAATAATTCAGTTGTATTTTATAATTAATATTTGGGGAGGGGGGCTCTGCGGGACTCGCAAA
ATTAGTTCTTCACTGTAATATCAGAGCAAATATACTATGCCAAGTTGTCGTGGGAAATTCGAGAAAGTCGAAGGCTTTTCTGCTACTTT
ATAATCTTCTTCGACATTCATTTTCCATTCATCTTTATAAAAACTTATTTCGTGTTTTCACTTCCGTGGCTCATAAGAAGCCGGGGTCA
CCGACCGGCGTGCAACAATTTCAAAGAAAACTCGCTGTCCACGTTTGTCCAATCGGATTCAGGGAAATCACACACAAAAAAACGGTATT
GTGCTATCTTGTTGTAAATTTTGTAAGTGACATTAAAGGATATCTTTGCACACTAAACCGCTTTCCAAACCATCAAAACCCAATTGGAT
TACCGTTATTCCAAGCTATAAACACAAATTGATCCAATTTAACACTTAAAATGTATACCCACCAGATGCTGTAAAAGAATTAACACGAC
CGTGGAGTTTTATTTTACAAGTCCAATTTGAGTCAAATAAAAGCCAAAATAACAGAGGAGATTTCGCGGGATTCGGCTGGTTTACAGTT
TTACAACTTCTTTTGGTTGTAATTTTGTCTGTGGTGGTGTGACACCCGCGCGCTTTTATTGCACGCTATTGCTTCTCGGACGCAACCCA
CAAGAGGCAATAAGTGAATAAAATTCAGAGGATATGCAAAGGGATCGTATATATGGGGATGGTAAACTGAAAGTGACAAAACTTGTATA
AAATTGTTGTGCCACGCGAGATATAATCAGTTGGCCAAATGGTACTGAAGTGATTGGTAGCTTTTTTTCTTCATGCGATGAGAATCTTA
TCAAGGAAGTTCGGTTCATGCTTACTGTTTTTCTTATACATGTATCAACATAATCAGCGCCAGGAATTATTGTTTTAACAGGTAAGGTG
TATGATTAACTGCTTATGGTTTTCTGAGAAGCATCTCATTGATTTGAACATGATGTTAGGATAATTTTCTTTAAAATGAAAACCCATTT
CATCAAGTCCTTTTTCCATTCTACACAATAAAGTATGGTTTGTCACGCGTGTTTTTCCATTAATGGCAGGAAATGATCTGCAGTGAAAG
CTAAATGTTTGTTTTTTTAACAACATTGTTCCGAGGTTGGTCGATAACAGAAGTTTTCTAATTTTTCAAATTTGCTATGATCTGTGGTT
TTCAAAACGGGTTTTCCGTCTTGAAGTACACTGAAGAAAGGGACAAATGTATAAGTTGAATATCAGTATTTTTATTTTTTGGAACAGCA
CCTTCTTTTTAAAGTTTGTTATTAAAATTCGTGGTTTTTTTGTTGGGGGAATTTCTGTCGGGAAAACATACTGGTTAAATTACTGCGTT
CGCAAACTCGCGACGTACTGGGACGACCATTTTCAGCCTTTCTTTTACTCCCCGGTTTTATGGGATGTTTTGCCGCAAATCGTAGTACA
GTGTACCACTTTTCTAATTCAATTTAGAGCGACTTCCAATGCGTTGACAAACTCGCGCCATTCGGGCCGGACTTCCGATCGCCATTCCC
TTATCAGTTGTTGACATTTGCCTCACACCTCCGGCTGAATTTTATCGCAAAAACGTCAAGATTTCGGCAAACTTTGTCCCAATTTTAAC
GACCCGTTCTGCGGTCACGGGCTGACCACCATGGGCGTGAGGGAGGGACCTTTACGGGGGAGTTCGCAGGCGAGCTGACGTATGACTCA
CGGTCGGATATCAATTACCACGGAAATGTTCCAGCAGCGTGTTACAATAGAATTACCGGATTACGGGACAATAGACTCAATACAGTCAC
CCAACTTCTATAAAAGTTGTGTTTGCCAAACATCCCTTATAACCCCCTACTGGTCCTTTCAAAACCCTCCTTTAGAAAAGCCGGATTAG
CGATGTTTCATTTTCAAGGGCGGCGAGATGAAACGAGCGGGGATGTCCATGTGGGTTTGCGTGGAGTTCTGGGGAATCTGCATGGGCTT
TTTTTCTCTGAGCATCTTTGAATGGAATTGTTTCTTGATCAGCTTTGAGAAACAAAGAGTTTTGGGAAAATTGGATTATGCAAATGCGA
TCGGCTAATTCGGTTTTTGCTCTGAAAGAAATGCGCAGAGTACTTTGCTGCGATGCGCTTGTCGTGAGGATCAATTGTGAAAACAAAAT
CTTCACGCTAACGCAGAAACACTGAACGATACTATATGCTTAAAATTATATTCCGGGTCGGAATTCATGAAAAAAATGGTTTGTACTAA
ATGATATATTTAAGGTGTTATTATCACAAAGTGCAGGCCAGCTAAAAAAATTAATCCATATATTCACATCGTGCCACCGTCACTGTGTG
TGCAAGATAAAAATGGTTTTCGTCTCAAAATGAGAAATCTTATTCGCCCAGTTTGTTAATTGTTGACAATTCTATACAATGAAAGCTCA
CTGTTAAAAAGCTTTCGGTAAGATGAATGCCTATAAGAGCGAAATACGAACCTTGTCATTTTATTGAAGTATCATATCATATATGAAAG
AACTACAATAATCCATTACACTAGCACCCTCAAAATTTGACTTGTATTATATTTTTATGGCGATTTCAGATCATATGATTCGGATTTGC
AGAGGTTGTGAAAAAAAGAAATTTTGAATAAACGTTAAACTTTGTTATGTTTTATAAACGCTAATGAATTCGCTATCAGCTCAGTATAC
ATGTAGTCATGTAGGTTGCCTTTGTCAGTAGTTTTTCAATAAATGTATCTTGGTTTGCTTGATTTTCCGACAAGGTAATCGTTTCTCAT
TCATCGCGATCATTCACGCAGCTGCAAAATGATTGATTAGTGTTCTCTGTATTACCATAGCAATGCATTCAATCTATAACTCCACGTTT
CTCACCTTTTACAGTTTGATTAACGAACATTCATAACTCGGTGCCGCTTGAGTAATATTGCATGACTGCTCGACAACCTAAGCATCTTA
GAAACTCAAAAATATAATGCTTGGCTGTGGTACATGGTTTTCCAGAAATGCAAATTTTTCATAAGACGCACGGAAAATGCAAACAATAG
AGGCTTACACGATGATGCTGAATTTTAAAAGAGCATTCAATCATTTTTATTTTAATAAAGTAGAAATTTTTGAAAAAAAGTGGAAAGCA
TGTAACAGAATCCACAACACAATGCACGGAGATTTGTGTGGATTTCAACCGCGTGACAGTAGTAAATTACTCTATAGTTTGCCGTTGCA
CCGCGGGCTAGCACCGCGTTCGTCTCTCGACCACACGGGGCCCGCGCGCTCCTCGCTCCCCGCCTCTGCCCCGAGTGCCTCCGCTCATC
GGCAACGGCGGAAGCCGGACATCGTAAATTTCAAATCGCGCCGATGATTCGGGGGGAAGTTCCCCCGACATCGGCGGAATAAATTGCCT
TCCCGGAACCCATTCGGTTACAAAAAGGACGGTTAACAAATCTTTCTATTTCATATACGGCACGGTGTAAAAAAAAATGACTCGGTATT
AAAAAAAAGACGACTATTTTTTTTGGTCACATTTTATTTTTTGATTAAAAGTTGCGAGTGTAATATTTCATCACAGGCGGTAATTTGCA
CGAAAATAAATGGCTCGCAGATAGCATTATTATTTGGAACCGTCTTTGAACAATGGAAATGAGTATTTCAGTTAGCAATTTAAAAATCG
GACTACGGCCGTGCCGATTAATTAAAGGCCTAACCTCGACAGAAATGGAAAAGGGACAAAGGTATTCACGGGACACATTGATGATTTAA
AGGGAATGTACATCATTGGTTTTTGCTGTAATTTTCAGAAACTAACTATTTGAGGGGATACTACAATAGATCAAATATTGTTTAACTGA
CATGTTTGAATGTTTGTGTTAAAATAATGCTGGAAACACCAAGAAAAACAAAACTGCTAAAGTACTCTGGTTCCCTGACGCCACTAAAG
ATGAAATGAATCAATTTATATATGCCACAAAATATTGTAAGGTAAGGGAACCAATCTAGGTGGCCTGCACCCGTACCCGTTCATGAACA
TGTAATCAATAGCAAACAAAATTTGGACTGAAAAAAAAAATGGTGAATTTGGAGCAGTAGTTGAAAAATAGTTAGGTTTTTCGGCCGACC
ACTGTTGGAAATTTATCCAAAAATACCACTTTTCTTGAAATTGACTTCACACCAGGTGCCATACTTTTACAGTATGCTTTGGACTATCA
TTTACCAACATCCTAGTAAGATATTTTGTAAAAATGTGGTAGTTTTGTTTTTCGATGTTTGCTAATGTTGTGTATTCCCTTTAATCACA
ATTTTACCTTTTTTGTCATACAGGT
**>*PmCdx* exon2**
AAAACTCGGACTAAGGATAAGTACCGTGTGGTGTACTCTGATCATCAGAGGCTTGAGCTGGAGAAGGAATTCATCTACAGTCGGTACAT
CACAATCAGACGCAAGGCGGAGCTCGCAGTGTCCCTGGGACTATCCGAGAGACAGGT

>**PmCdx exon2**
KTRTKDKYRVVYSDHQRLELEKEFIYSRYITIRRKAELAVSLGLSERQ
**>*PmCdx* intron2**
GAGCAGCAAGAAAACACCGGGAAATAAGGGGCTCTATTGTAAAATATGGACTAAGAAAAAGTCCATCTTATTCCTCCCGATGATTGATA
TCACATTACCTAAATTGTTGATTATACACATTTCTGGGAAGAAATTGGCAAAAGTGTACTGCTTCATGCAGTATTATTAAACCAACTCT
GGTGCAAGAAGCCAAACTGCTGGTTCTGTCCGTATTTTGTGCACAGGGGGACATTATTACCAGTGCAATGTATATTTTTTGGTGTCGGG
GGGGGGGGTTCCGCGATTATTTTTTTATGAATTTCTATGAAAACAAATCATTTTGACGGAGCTGGGGGGGTTGACCGCACCTGAGAGTA
GACAAAGACAGATTTTTCTTGTTTTTTTTTACCGACACTTGGCCACTCAAAATATGCACTATTATAAATTTCAAGTACACTTTTAGTAA
GTCTTTATATTTCGAAGATCGGCAAATTATGTTATCTAAATCCCTCTTTCGACTTGTGAAAATACAATACTTTCTACGGGCAAGTGAAA
CAGTAAAATGGCACTAGCGCCCTCAATGCCGAATCATGTTCATGGTTGTTCAACGATACATGCAGGCAAAAATGGTAATTTTTGTTAAA
ACCAAAAACAAAATATAATGCCAACTTTAGTGAGGGGAAATGGGTCACCCAAACTTTCATATTACCGGTAAGCTCTCTCTGATGTCATG
TATAAAATAAAACCCCAAAATATTATCGATATTGTGACTGAATAATTAAAAAGGAGGATGTACATGTAGTCTCTTACTTTTATATTTCT
ACTCTCCTATGATGCATGTACATGTGTACATGTATTCAATTCGACGGCCACACCTCCGCCATTCGCTCTTTTTATATACACAATCAAAA
CTTTGCTTATCTTATTTCCCTAACTTAGAAAACCGCTTTAGTTTTAGATTGTAATACTGTTTGAAGGAATTAAAGAAGAACCATTGCAA
TAGAAACACTCGATCTAATCAATGGACTGAATTTCACTATATTAAACGTCTGGTGCCTGTATCAGTCGGTGGGAGGAAATTTCTACCAA
AATACAGTAAGTTTTCACTCTTTGAAGTTCTTTGCTCACGACACAAACATTCTCAAATGTCAACTACAGGT
**>*PmCdx* exon3**
CAAGATCTGGTTCCAGAACCGGCGAGCCAAGGAGCGCAAACAGAGCAAGAAGCGGGCGGCCCTGAGCGGCGAGACCACGGCCGACCAGA
CCCGGCTGGACGCCTCCCCGGACGGCGCAGTGGAAGCGGACAGAGGAGGGCCGGACATCAAGAAAGAGGCGGCGATCAAGGAGGAGTTG
GACCCGGTCGAGACGGGGGAACGGACCCCGGTCAGCACGCCCGAGGTGCTGGAGATTGTCCGCACCGCCAACCCTTGCTTACACAACAC
CACGCACCCACCGCCGGAGATGGTCCTGAACGCAGCGGTGCAGATGCAGCTAGGTGGTACCGGAGGATGCCACCTTCAGACGGCCCACG
CGACGGCCACCGCCTCGCTCCTTCACGCCATGCCCGCCATGCATAACATGAAATGA
>**PmCdx exon3**
VKIWFQNRRAKERKQSKKRAALSGETTADQTRLDASPDGAVEADRGGPDIKKEAAIKEELDPVETGERTPVSTPEVLEIVRTANPCLHN
TTHPPPEMVLNAAVQMQLGGTGGCHLQTAHATATASLLHAMPAMHNMK

## PmLox:

**>*PmLox* exon1**
ATGATGGAGGGTAACAGCCCATCGTTTTACAACTCGCCCTTCGGTTTCCACAATGACCAGTTCGTGGGTCCGCCCCTCTCGGGCGGCAC
GGTGCCCCACCGGAGCTACAGCCCGCCTGCCCCGCCGGCCTGCATCTACGCCGACAAGCCCGGCCCGCAGGTAGGGGGTTACGGCGGGG
CACCCATGGCCGTGGTGGAGTCCCGGGTGGACGAGGGCTTGCTGCAGTACCCTCGCATGACCAACATGAACATGATGACCCTGGCCAAC
ATGACACTGCCTCAGGACACGGCCTGTACGCAGGCGGCTTGCGGGGCCGGGACTGGAGCCGCGGCCCAGCCGTGCGACGGGGTCCGGGA
GCCCATGCAGGCCGGCCAGCTAGTTCACGCCGTCATGCAAGGCACCACGGCGGTAGCAGCTTGCCGAGGATCGAACCAGAACCTTGGCG
GTAACCCAGGGCCGCATCATCATCATACACCCACCGCCTCGGCCGGCAAGACCAAGGCCGAGGATAGAAATCAAAACCTTCCCTTTCCA
TGGATGAAAACCACCAAATCGCACGCTCATATGTGGAAAGCTAACTGGCCAGG
>**PmLox exon1**
MMEGNSPSFYNSPFGFHNDQFVGPPLSGGTVPHRSYSPPAPPACIYADKPGPQVGGYGGAPMAVVESRVDEGLLQYPRMTNMNMMTLAN
MTLPQDTACTQAACGAGTGAAAQPCDGVREPMQAGQLVHAVMQGTTAVAACRGSNQNLGGNPGPHHHHTPTASAGKTKAEDRNQNLPFP
WMKTTKSHAHMWKANWP
**>*PmLox* intron1**
TAAATCCACAATCTTCATCTATTCGTCGATGTTTTGATTTTGTCATCTTATCAGTTCATTAACAGTGGAGTAATTCCCTTACACGGACC
TGTCATTGTTTTGTTTCGTCGTTTGATCTCATTAAGAAGCAAATATCCTAGCATGCATGTTTCATCAAGAAAGTCTGGAGAGACATTCG
CATTCGTACTTTATTATCATTCCCAATTATGCACAACTCTCAGAGCAAAACCATTTAAAAAATTGAATCAATAAAAAAGACAGAAATAT
GCAGTCTGTTAATTAAAATGAGCTTGGTTCGTGGTAATCGCGCATGCTGCATGGCGGGACGTATACTAACATATTACAAACATTTCTGT
TGTACTTTATTAAAGTGATGTTTGGTGTAATCCAGACAAATCGAAACTCCTTTAAATTTTCTTGCATGCCATATAAAAAAAGACACTCT
ACATCTGATTTGTCAAGGAATAATGATACTCTTCACAAAACCAGATTTGTAAAAACATCCAGTACAGCTCAAAACATGCACACGCTTTT
ATATTCTTCGGCAAATGAAAACCTAAACAAATTGACCCCTTTTTGAAAACACGTAAAAGCTGTAAACTTCCCCAGTTAGACATTATGAG
TGCCATGTACCATTAAAGCAAGTCAGCGTATACATTATCTGTCGTTCACAGAGCACTTTGTAAATTTGACGTATTTTTTTCATTAGTTT
TCTATTTATTTCTCAATAACTTGGCGTCATTCCTCGTATCAGACATGTAGCGATATCGTAATCTCGCCAATCGCTCCGTACCACAATAT
TACTGTCGCTGTCGCCTTGATTAACAACCGTCTAGCTATTGTTGCAATTCACTCAAATGCGACCGACATTTCCCAAGCGTTTTGTTATC
TAACTACAACCTCCCAGATTTTTGTAACAGAATTATCTGACGCAGATTTGGAAAGAAAATAAAAATAGACTTTAATTGATGGATTATAT
ATAAATTCAGATTTGATTTTGATGTAAATACTCGAGAATACAATTGAAACCATTTTTCAATATAGCCAATTAATTTTCAGCGAAACAAT
TTCAGATATTTTATAACTGATCTTTGAAATTTATGTCACAAATCATCATAATACACGTAGGCCTAATACCCTCAAACGCTGACGAATAC
AGTTGTCATTCGCCCACCAATGGATTTGTGATGTTAATAGAATTGTGTCTATCATTGTTTTGCTATCGTGTTTGTGGTTGTATATCATG
TTTGCTTTCTCCATGTCCATGCATGTTTCGTCGGTCGTGTGCAGAACATTCTACATATGCGCTTGATCCTCAGACGTGATAGTTCCCAC
GCTAAAATAAAACTATTCTATTTTTGTCATTCTCATACCGACATTGCCGTATCCTGATGACCGGGTTGACTTTTCTGGGTCACTGAATT
TGTTTACTTCAAAATTGGTGATTTCTAGGAACAACATAGGAAACCACTAAAATAATATTTCATGCATGCCTTTGTGTCTTTGACTTGTG
TGTTTTTTGTAATTTTGGCTTTGACAAAGATCCTTCTATAATTATAGGAAGCGTTAGGCCCCTTAATATTTTCTTATTGGAATAATTGT
TTTCCTTACAAAATGCAAACTTGACTTTGCCATAAATTGAGCAGATAATCTTGTACATAATTTAGGGTCCTTTGAATAGTTAAGAACGT
TATTTTGGAATAAAAATCATTCCTCCCACTATATATCCCAAAATACACCAAGTGGGTAAAAAAAACCGAAGGTGAAGTAGGCCTATTCC
GCGATAGGAAATTACTGAGCGGACCGGCAGCGCGTGCTAACCCCGACCGGCACGGAGATGACACCCACGGTCACGCGGTCAGATTCTCG
GTAGATAGCTTCGCGTCATCGTGCCAAGCTCGTTGTTCAAAGCTCCCATGTCCATAAAACCGAAGCATTAATAGGAACACAAATTACAT
GTCAACTCCTGATGGTGCAATTTTTGGCAAGGAAGGTTAATTTTTTATACTCAAAGACATGTTTAAATTGGTAATATTTTCTGAAAAGG
ATGTGTGGTAATCTTAACGTATCAATCAAGGTGAGGAGATAAAATCTCTATTACATTGCATTCATGTGATTAGTCTTCGTACCCTACTG
TGTTGGAATGTGATTTAATGCACAAAAGCTACCGCAAGAAATAGAATTAAGCTGTATACCACAGTTCGCTTATTTCGCAGGAAATGTTC
AATGATTTCCTTTCACATTTCAAAAATTGAATTACAATTGCCTTGCTTTAATTGCAGCATTTTGTTTATGATTTCACGCTCGTTTTGGG
GTTCGCATATTTGATGTAGGCCCCATATTATGTTGGTGATCTTTGCTCATTTTATCATGCAGTCTGCTAATGCATTTGGAGCCTAGTTT
TCGATACTTTTCAGCCAACCCTCATTATCATTCTGTATGAGCTTCCCTTTCCTGTTATATTCCGGAAACTTAGGCCCTGTCATATCTAT
ATAATTATGAAAAACATATATCGCTTTCAGCAAATTCCCCTTTGTATTCTTTCAGAGATATGTCAATATGCATGCAAAAAATACAGTAA
AGTACTTTTTATTCAGTAGAGTCACGGTGGTTTAAACACTTACGATTTGCTGACATATTTTACAAATTTGTTTTTTTTTCTGAAATTCC

AATTGATTTGTTTATCTGGAAATAAACCACCAATACGCGAAAATCAAACTTGTCATTTTAGATAACTGATTTCACTTAACTCAGTTCAG
CATAAAGACGACGTGAAAATATTTAGGTTTTGTTCTTAAAACGGTCACAAATTACTAGGAAAGTGTGATTTAAAATAGTGATTTCGAAA
TCTCAATCGCATATTTCTTAAACATATCAGTTTTTTTTAAACCTAAGGCTTGTTTCGATTTTTTGTGGAAGTACGTAATAGTTCCTGTA
ATAAGCAAATAGAGAAAATACGTACAAAATAAATATAAAGAATAAGAAATAATTGATTATTTCAAACTTAGGCAAACCACTGAGAAATA
ATATTGAAAATATTGAGGCATATCCGCAAGTTGATTTAATTCAAAATTAATGTATTCGGGCCGGCATGCGCGCCGCCGCACTCACTTAT
ACTGATTAATATACAGTCTCGCTCTGAACTCCATTTTTTTTGGTAAATTTTCTCGGCACTGTCTCACAAGTTGTCATCGATCTTGACCG
ATCGATATTTTTGTTAAATAGGGGGAGGGGATAAGGGGGGGTCATGATTATCAATGTGTGCCCTGCTTACTGGGCCTACTTGAGCCCGA
CAAAGGGCCGACGAGACTTGTATGCACCCCAGAAGTGATAAACAAACCAAGTTTGAATTTCAAATTTGCCATCGGTCAACACACAAACT
GCACGGGTAAAGTTGATGTTTTGAGAAGTTCATTTTTCTTTTTCTTTTCCCCTCTATTTCTGGATTTTCCGGCATAAATATCCCTAGCT
TTAAAGATAATCGTCTGCAGCGATAGGGCGAAGGAACCTAGATGTCCAAGAAAAAGAAGTAGTAATACATTTTGTCGCTCAGGCTTCAA
AAACAGACTTATAGGCCTATTCATGACGGTATCGAAATATGACAAAGCATAGAATCAAAATACATGTAGGCCTACATAAAACTATACAA
CTCAAAAAGGATATGAAACATGACTATATTATCCATTCCCCTGTTAGACAAAACATAATTTCCCAAATGCTCGTTAGATTTTCTCGAGT
TTGTTGATTAACTTAGATAAGGGAGTTTCAAGCTTATATGGCCCGTGACCGAGTTGCTTGGCACCATTTTGTTCGTCTTGTTCATTCAT
TTGTCAGTCTGGTTCCCAGTGCTATCCGGAAAAAAAAAGTTTGCTCCTGAGCAACAATACCTGTAACTGTTTTTGTTGCCTATTCAGCCA
TGCATGTAAGAGGGATCTAAAAGAACAAGAGACTTTATCATGATCCTCAAAAGTCCTGGTGGGCAGAGTTTCTTCCTTCATCTTCTTCT
CCTTTTTACCTCCGTCTTTTGAGATGAGGTTTAACCCAGTCATCAAGTGTATTTGTTGAAGTAATGATGAGCGTACATTGTTACCATCA
TCACCAGGGGAGCACGGGCGGCCCCTTTTTGAGGATCGACTACCGGGTCCGACATTGATTAGTGGGTTCTTCCAATTATCTGGAGTCTG
GGGAGAGTGAATAAAACTCGCCCATAGCTTGAAAGATGGGGTACTTTTCGGATGACAGACTCTGGAGTCATTGACCGTGTAAAGGGGTT
CTGAAATACCGAGTGAAAGACGCCTAAACGGGTCCTCAGATGCCCAGGAAAACCTGGACGCCTGTTCGTTTTAGAGAAAGTCTGTTCTT
TTGTAATGATGTAATGAACTATGATACAAATCAAATAGAACAGACTTTAAGATTGATTAGTTTTTCCCCTATATGTAAATAATAATGAA
AGCAAACTGCCAAACGACAGTAGTTTTCGAGAGAAAAAATCCACTGTCTCTTTTGGAGAAAACAAAATCATGTAACAGTTGCGTAATGA
GTTTTGTCCTTAGCTCAGAATGCTGTATTTATTGGTACGCCATTTTCATCCATGTTTCTGGTAAATTGACATTGCAATATATAAAAGTG
CTTTCTTTTCACCAAAGAGACCGTGACAGCATAACAATGGACAAAATTATCGACATATAATAAGTCACTTTTCAAAAAGCTTTTTTGGG
GGACAAATTCGTTGTGTGACATGCCACCTAAAATGCAAAGCCAGTGATTCGTGCGCCTTCTTTTGTTTCTTTTGCGCGCCATGCATGAT
AAGGCCATCACAAACCTGGTCAAATTTGTCGTCGTCTTTTGTCGGGGAATTGGAAAAGTATAGATTTTGGAGAACTGTCCGTAAAACAT
TAGCAATCGTAACTCTTTAGGAACAGTTAACTGCAAAATAGAATATATGGCACACAGCTAATAAACTAATACCAACACAATGTGGCTAA
CTTAGCTTGCATTTCGCACTCAAATTCAAACAACAAAAAATGAGAGATTTGAGATAAATATAATATCGCAAAAGTGAGGTATTTGCACG
CATAGGCCTACGTCAAGTTTTGACTGTGTGTCGCTACCGATTTCGTTGACAGGATCCATGCTGCAGGTTTTATCTGCTCACAAAGTGTC
ATTAAGTCCTTGATTACAAGTCATTAGTCTGGTGTGTGACGAAATAAACCACACGAACAGTCCTATGCAAATCTTCAGACTGGGGCAAT
TGAATTTGGCTTTCGGTAAGAAAAGATAATTATAATCAATGCTTTAATATAGATCAGAGCAAACAGCGATCATGCTTTGATTTTTCTGT
TTATTTATATTTGGGATAGATCAAGATGAACTTGGCTAATTGAGGTCGGGTTAGAACATTTGACTGTATAGTGTCTCCTGCACATGAAA
ATGTTTGGGTAGTAAAGAGTTGTACGTTTTTGTGCTAACCAATAAAAAATGTCCATGATCAGTAGCTGGACCGGTTCCAGACAGGCCGC
ACGCCGTGTAAAATCTACCATAGCTTCTCCGCTAGTGTCCCACCGACGCTTAGTCCAAAACTCTCCTTGCGAAGGGTTCCCTTCCCCCG
GTAGACCACAGACCTCCGGAAAACAACTCAGCTGAACAGGTAATGAGGATCGGACGACCAACGAATTGGTCTGTCCTTTTGCTGACAAC
GACCAGGAGCCGGCCGCGTGCTCAGGAGCCAAGCCCCCCGGGCGATTGCGCGAATCCGTCCCGAGGCGAATTGCGCGCTTCCGCGCTCA
TCGATCAACTCCATTAGAACTGGGTTTTCCCCTACAATGCTACAATACGACATCACATCTGGCCCACTGATGCGCAATGTGAGTCGGCA
CAAACTCAAAATCTTTCTTGTTTCTGTATGTTAACCACCGAGCTTGAGCTTCGAACGATCGTCTTCCATCAAAGAAGTAAAAGGAATGA
AAGAAAATATCCCCGTAATTGCGTCTCGCTCGTTCAAGTTTAAACCGGGCGATCCGTCAAAGTCTCGTGGGTGGTTTGAAGCCGAGACA
CGGCGGGTCGAGACTGCGTGCGTCGCGCTCGGCTCTTATCGTGGAATACCTCCTTGGAGTCTCTAGCACCACATCAGTCTTACGGGATT
TCTCCCTATCCCTGTCATGTCACGCAGGCTATAGCGGAAGAAACATTTGCAGACAGAGGCAAACCTTCAGTATGCTGTTTTATGACTTG
AGACCCGACATAGTCGCTGACTGGCTCTTTTTTTGTTTTTAAATTAATGACTTCTCATGTCTTGGTATCACCTACGCGAGGTGTGACGT
GAGTTAATTGTAGCGCAATGCATCACCTGTGTCTCACTGCCAGTGTAAACTCTTTCTCATGTAGGACATACACAATATTTATTCGCTGT
CATCAGGCAACGTACGCGGTCTGCTCTTGGAGTCCATGAGCTACCAATGTTGACAGTAAACCCAACTCCTTTGTTTCTTTCATTCTTTT
CCTTCTTTTCCGGGTAAATTCCGAAAATTTGGAATTTGTTTGTTGGTTTGTCACTCCTTCGGTTTATCCACTTGTCTACTTCCATATTT
GTATTCCAATCTCCCTCTGGATGTTGTACATTTACCTGGTGGCTCTTTACATCTTCAGTTATGTACCTTTTTCCTCTATTGAAGTGCCA
CAGTTAAGGTTTCGCATAATTACATATTGTTCAATAGCCAATTTTAAACATGTATCGTTTAAAAAGAGCCGAAGTTTCTTTTTTTTCTGA
TATAGGAGCCACGCATTGCGTGAGTAGTTATAAGCGACGCGCACATCCCCAATATCGAAACCCCAAGGTTCGTTTTGTTCATAGCAACT
CTTTTGAGTCAACTTTAAATTTTAAACATTTGTCTTTCTCTTTTAGCTTGATTGAATTTCCTAAAAGTATTCCCGGATCTTCAGATGGG
CGTCTATTCTGCCAACAAAACAATTACCCAAATATCACCCCCAAAACAAACAAACAAGCAAACAAACAAACAAAGTAATCATTCTGAAT
ATTAACCGGATCTACTCTTCAGTCATTTAAAATCCCCAGTATTTTGCAGTCTGCATTATATATTTGTTGTGCGAGTCAATTTGCACAGA
ACAAATTGTCGGCAAACTGTCAAAAAAATGATAATTCTAAAAAAAAGTATCCTGTTTCCAGAGCGCTTGCCCATGCTACACAACCCTTA
TTCATAATCCTCTGCGCCCAATATCCACACTACCTCCCCCATCTCTCCATCACTTCATCTCTCATACCCAATCAATTTCATTTACTCCA
AAAGATGTAACAACAAAATCCCCACACTGCACTTCTTCCCCTCAAAGTTTCTCTATGACTGGTTTACGGAATTTACTCCCGCCATAAAC
CATCCCCGAATGGCCCTTGTCGTGTCACCGAGGGCAGATTATAGCAACATTTCCCCTGCAGTGCTTATTTAGTGATTACAAGACCACTC
TGCAGATGTATTTGCTTGATGCGTTTATGGCAGCCATAACGCAGCACAAAAAGCCACAAAAGGACTGCACTCCAATGGTACACGCGTAG
TTGAACCCCTATTTTCCGATACCGCGAATTGTAGTTCTCTTGTCGTTTCAGATAAGGTCCGAAGTTTCAGATCTTGTTGATAATGAGAA
CACAAGGGTTAATTTTTTGCTTATTGATGTGTAGTTTAGAGTTTGTGCTTCGTGTCGAACAATATCAGACAATGGTGAAACATCAGGAC
CAACTAAAAATGTTAAGAGCAGAAAATATTTGCTGAGTGAAACCAAATGATGAATTTTACATAATATTTAAATTTGCTGTTAGCATGTA
AATTTGCCATCGCATTGACAATAGGTTTCAAATATGCCATAGCATGAAATTTGCCATTAGCATCTAAATTTGCAATAGCATTTAATCTG
CCAATAGTAGTCCTATCTAAGTTTGCCATGGCATTTAATATTGCCATTTGCATGTTAATTGCCACAGCATTTATTATTGCCAATAGCAT
ATATATTTGCCATAGCATCTAAATTTGCCATAGCATCTACATTTGCCAAAGCATCTAAATTTGCCATAGCATCTACATTTGCCATAGCA
TCTAAATTTGCCATAACATCTCAATTTGCCATGAGCATCTTAATGTGCCATTAGAATCTAAATTTGCCATAGCGTTTAAATTTGCCATT
AGCGTCTAAATTTGTCGTGGCATCAAAATTTGCTGTAATATTTAATTTTGTTTAATTTTGCAATTAACATCTTAATTTGCCCTAACATT
TAATATTAATAATAGCTTTGAAATTTGCCACAGCATGTAATTTTGCCATAGCATTAGACATAAAAAAAATTCTGCTTAGCCGTTCCCGG
ATATAACGAGACTTTGCACAAAATCCTGGGTCGGAATGTAGAAACGGTGTTGAAATTAATGCGGTCTTGCTTAATGGGGTTGTGCTGTG
GTTAAAACTGATAAAACTAACCAAAGAGATTGTGTCAGGTCCTTATTAATTGGTGTTAATTGTAATGTGCTTAATTATCCTCGAATTTA
ATCACTGCGCGTATTGTCGGAATCTAATGAATTGTTTATTGTGATACATTGTAACAATGATCAATATACATTATTATATTATACATCTA
TATACAATTATCAATATCTATATACGTATACACACTTGTCAATATTGAGACAAAACCCAAATGCACAGATAAGCGATGTAAGAGTTTTA
AATTTGTCACTGTCTGCAGTTCAATACTTTGCATATAACTGTCAAAACATTCCACGAGCCATTTCACGGATACTGCCATCTTTGTTGTC
ACGTGGTAACCTCAGGGAGGCCTTTCAATAGAAATACGTCCCATCATGTTCAGGATACAACGGCATGGTGGGACGTATTTCTATTGAAA
TGCCCCCCGGAGATTACCACATGACCAAACAGATGGCGGTATCCGTGAAACGGCTTGTGGACAATATCCGAGTTTTAATTGTTTTTTGA

ACAATATTTTCAGCAATGGGCCTCAGGATTCTTCAAAGATGATATATTTGAAATATTCAATAGGCCTAGAGGTTTTACGTGGCATAATC
TTCCCATCACATTGCTTTTCTGAAGTCGTAAGAACAATAAAACTTGTCAGTGCCCCTCCATGGAAGAAAGCAAAAAGGAAAAAAGGAAG
ACAAAAACTTAAACAACAAAAAAGAAAACCTAGTAAAAAAGTCCTTCAGTTTAAAAGCTGGTACTCAAAATCGTCCCTTCGTTTTAAGA
CAAATACCTTTGTACTCAACACCATTATAGATACCATTACTACTACTTGTTCTTCTTTTTGAATATTCTTTATACTTCCCTTGTTTTAT
CAGTCTGCAAAGTTATTTCAATTTGTTGAAGCTGGCTTATACTGACTGCTAACCCAAGCCCATATGATCCTGTACAGCTGTTGACCTGA
ACATGCCAGCCGGAAAAGTGTATATAGGCATATCCGAAAGTCCTTTATTTTGCAATTGTGGGCTAATGACAAGGCCCTCGACAGATTGT
CCAAATACTGGGACACAGATGTAAATATCCATTAGTATAAATTTATTTTAGTTCGGTTGACTGGAATTGGTTTCGTTTTGGGTGCTTGT
AATATTTTGCTGTATCTCCGTGCGTTACAGGTTGCAGTTGATGTGAAGCAGTAGTCATTACCAATGTGAATGATAACTTTACTTGTTGC
CTAAGTTTTGGTCTTTCTACGTTGCTCTATAATTCCTATCCCGTATAGTACAGAATCCATCTACAAAACAGTAAACTTGAGCAACTTGC
CTCTTCGATGCAGGCAATCTCCGAAGAAATGAGCTCCCAAATTTATGAGGATGAGAGAAGAATGAGTACCAGTGCTTGTTCCACTGGTA
CCTGGCAGCATGGCGCTAAAAGGTTGATTTTTCAAGAGCTTCGTTCGGTCACTTAGGTTTCGTAGGCATTTTTCCAATGGCGGCACTCA
AATAAATTTAACTTGGGATATGCATATTGAGTTAACCGTGTGATTTTAAAATGTATCAATATTATTCTTTAACATATTACTATCATCCG
TTTATCAGGCTAGTGTTGCCATCACGAACCAAACTAAGTTCGGTTATTTTTTACCTTGAAGCATCGCAAAGTTTGCACGCTATCTATCC
AAATTACGGTGAGCCGTACCGGTATAGGTTTATGCGGGTTGTGCACCCATTCTAATGGAAATACTAATTATTCCGTATTGATGAGTTCA
GGATTGATCAACGTAAACGCTTATCTGAAATGATATGCTCCATGAATATGTCCGCGAAACAGGGACAAGACGACCAGATACCGTGGCGC
TGCGGGTGAGAAATCAATGGCTGAGCATGCTCACCATTGAGTAAACATGGCTAAGCAGCGTAAAGAAGAGTTAAGTTCGTTTTAAACCC
AAAAAAATGGAAAAATAACTACAGATTTGGCGTTTGTGTTTGTGACAAACGAAGGGTGATGGGGCGCGACCTATTCGGTTGTTTGTTGT
TTCAGTTCACGGTAACGAACGTCAATGTCAAATGATAATGATATCATTCCTGATGCAATTGTTTGCGTGCAGTAGACTGTTTCGCCTCG
TTGTAGGTTGATGTTAACTAGAAATGGTTCACCATTTAGTAAACATAGAATACAGCACTTCAAAAAGTTGAGTTCTGTATAAAACTAAA
GTATCATTTGTAGTACGTATTTAAAGAATGAGGACACACTGTTTAACAAGTAGACATCAAACCAGTATTGCTTCCTTGTCGTTTCGGAT
CGAATTTCATCTACAGTCGCAGCCTGATCATGTGAGTTTATCCCCACTGATGGCTGATCCCACTTCTGCAACCAAACGCACATTAATGT
TGATATGTACACAGGTCTATCTCGATAGTAATTCGTTATTTCTCCCTATTCGCAGTGTTCTTTTTGTAGAACTTTTTTCCCCAAAAGCT
CCGGAAATAGGAATTTATCAATAAGGGTCGGGTGCCAGATTTCAGCTTCGGAAAGATTGAGTCACCGGCGTGAATCTTTGTGATGAATC
ATTATTTTAAAACGGAAAAAAATCAACCTAATTGTAAATATAATCTCCAGACCCTGCAGTGTGTTCTCTTTTTTTCGAGTGATGACCGA
TCTTTGAACGCCTGTTTAGCTCCCGTATATCGGCAAGCCAGCTCTTGCTTGTGGTTCGATATGATAGGGGTTATCTCGGACCGGGACTT
GTTTCGCTTTTGTTTAACCTGTAGCAATACATGGCACCCGATGGCCCCCTGGTACAGCTGCTAGCCTGTCGGCCTAGTGATAAGGCCCT
TTTTACGACTTTTTTCCAGCAACCCCTCAGTGTATTCTATATATGCCGACGAGTGTGTGTGTATAGCTTGTCTTAGTGATTGCAGGAAG
GCATTGTATGAAGTACCAACATGCGGGCCAAAGTCTCCGGCAGATTTTTGCCGTTGATAAAAAGTGCGAAAGTTCAACACGGAAGAACA
CAAAAAAATATGTATGGCACCCAGTCTTTTTTTGGACAGTCTTCTCTTATATCTAACGATCAGCTCCAAACCGAAGTAAGTGACTCTGA
CCCGAGGAAAGTGTTGTTTTTCCTGATGGGTAGGCTGTATAGACAGCATGAACCTCTGAAAAAAATGTATCTGTGTACATATCTTACGT
TCAAGATTCTCCAAGAACATCACCCTCAAAGTGAATGACAAAAACTGAAGGCCTGTTTCAAGAATCGCAACAAAAGAATCTCCCACAGA
AAACAAAACAAGATTCAAAAATTGAACGATCCTTTTGTTCTTATTGTCGTTCTTCTTAATCTTCTTCCCGTAATTATTAATTTTATAAA
CCAACACGTAACAAATAGCAAACACCCAAGAGACAAAACGATTAAACATCAAATATCTTTTGATAGTCTTATTTCGTTTAAAAAAAAAT
AACGGTCAAAGTTTGTCGGTGAGCATCCGAAAACTTGTTTAGACACAGATCAATTTCAATTACGGACCAATTCCTTATCAAAATAAATG
TTTTGAATTGAATTCTAGTCTTTCGTGTTTACACCGGAAGAACCACCCAGTCCTGCCCCGTTCGTTAGCCTCAAGGTCATGGCACAAAC
AAGTCGCATAACTCCGCACAATCAGAAAGAAAATTGGCTCCCATTTTTATTACCGCGTGAAATTGCCGAGTTATAGAAGTGCCATCTAT
CGAAGGCTTAACTGCCCATTACTCAACTATGCACGGGGCGGAATAATGAGCTTTCAAAATGTTACCTTCACAATTAGAAATCTATCAGT
TTTTTGTAGTGGGATCAAGAGGGTTTTCCTATTTTTGTATTCCTTAGGTGTTGTTTCCGCGATTTCTTATTATTCACTTGTCCAAGTTG
TTGACATAGTCGTCTATGTCCTCCATAATGAAAGGAGCTTCAGCATGAACTAAAAAAACTTTGTGTGTTTGGTATAAACATCTCAGTGA
GGATCACTCTAAAATTATTGTCCAACAGTTGCGATAGGTTTGTTTCTTATGACAAGACTGTGTCTTGAAATCGCAGACACTGTTTCGCC
ACAATAAGATCACGGGTCCCAACTTCAGTTGGGACGGGCCCAGCTTAAGTTGGGCCCGTCCTAACTATACTTGGGACGGTCCCAAGTTG
CGACCGATATTGTTGGGGCGTAACCATACCAAAGCGCAATAGGTCAGCTTACTGCTACCCATTGAGATGTATTACTGACATTTTCACGG
GTTCTTTGTTTTACATGAACAACGTGGAACAGCAAACACGACTTTTTTTACTGGCACACTAGTCATGTTCATTTGTCCTGCAATAACAT
GCATACCAAAGTTAGATCACGTCATAATCTTTGCTATGACTTGACGTCCATCTGCCCATCTGTTTATTTCGTTGTTTTTTCTGTGGTCA
TGTTGTTTGTAATAATTGATACAGCAACGCCAGATGATGGACATGCATCCAGTTTCATTTTTGCTAATTTGGACTTTCGATACGGTAAT
CGTCCTTTGTTTGGGGTTATTTGTTAATTTGTAATTGCTTATTGATTATTGTTTACATCTTCTGTGAACTCAAAGTAAATTGTTTAATC
AAGGGTTGTAGCTTTGCAGCCTGTGTGGTTGTGTGGTAAAATTTAATCAATTATTAAACCGCAATCACATAAAATATTTAAGGCTAAAC
TATTTTGAATAATTTTGTCTTACAAAAAACTCATTTTAAAAACAACGGATAAGGTGCCACAGGGTTCGGCACAATTGTCTTAGAAGGTA
AACAAAGATGCTGATAGAACTTGACAAACTGTTCTTTCAAAATTGCACAAGCGTAATTTCAGGGGAGACGTATGCAATCTCGCAATGTG
GTACCGCCCCCAGATCATAAATTTTCTGTAGCTCCGCGGCGCTGCAAATTGTCTAGGTATTTTAATGGTCTTTATCTTAATGGGGGCCG
GTGTTCAAATTATATTCGGCGCGGTAACACAGCCACTTCGTTACCGGGCAAACTGACCATTCAGGATAATCAATTTTGTTGTTACACTC
TAAGAGTGAGCTGAAATCGATGGAAACATTCAAAAGTAGCTTTTGTTTGCGCGCAAAGAGGCAAAATTACCCTTTTGGCATTTTTGATT
CAAGGAAAATAATGCGCGTAAAGAAAAACCGGGGTGTTAATTAAACCGATATTACCCGTTTTTGGGGGTGCATTTATTTCAAAAATACA
TTTACAAGAAAACCGGAACATGGTGTTTCAAACCTTGTCATGTAATTAAAAAAAAAATCTTGCTTGAAATGCGATAATAATTTGTTTCA
ATTTGTGAGGGTTTTTTTTTTATTAATGAAATGTCAATTCAAAAAGGCAGACATTTTGAATTAGTAAAACTGCGATTGGATCTTTGGGG
TTTCATGGTGTTTCATGTTGTGGGTGCTTAATTGAAACTCATTCAAAGGCATTTTTTGGTCTTAATGGGGTTTCAAAACATTTTTTTTC
AAACTTTTTTGTTACAATTCTCGTTGACTTTACACGTGTTTTTGTTAAGGAAGATTCAACTGAAATATCGGCTTTGTGCTCAGTTATTG
AGGATTTAAATGAAGTTTTGAAAAAGTATTGATATTCGCCAGTTTACAAAGTAAACCGGATGATAAAATATACAAGGTGCAAACTTCAG
CTCCGCATGTTATTGCATGTTAATCATGAAACAACTTGGTAGTATTGACAAGTTTTCAATCTGCTGATGCTATTCCGATAAAATGTGGC
TTGGTACAATAAAAAAAAAAATCCCACGAAACTAATTGCCATTGATAGACTCAGCATTTCCGACTGTGTGATTCAACGCACCGTATGAG
ACCGCATCAAACGTCACCAGACTACAAAGCTTGAAACTACGGGTATAAAAGCCCCCCTCTATGTAACCCCCCCCCCCTTGCCCCTTCCC
GACTCATTCAATTCCTCCAGTAAGCCAAATCGTTCAGTTGGTTTTTCAAATTCGTTAACCAAACAACTACAACAAAGCCTGCCAATGGG
CGCCGAATTAAATAGAAGCACCCACCGTTTGTGGAGTCAAAAACTTCGCCCAACCCGTACGGTGTATGGGCCGAGTATTCTATTATAGC
CCCAGCGTGGTCTGTTTGTTGTGTTGTCTTGTGTCCAATAAGCCGCTTACACAAACACTCGGACCGGGTTCATTTAGCTGGGCTTTTGT
TGGCCAGATCGTTGGTCCTGAATAGGGATGTAACCGATATCCACTATTAGATGTCCCTTTTGGGTCTTGTCTCTTTTCTCCGCACGGGG
GACTTCAGCTCTGCAAGCCCCCATGCATGTACATGCTTGAGTGGGTCTTTAGGTGCCACGAGATGATCTACACTAAAGTACATGTACCT
CTCTATCTAGAAGGGGGGCAGTGTTGGTTTTTGAGGATCAATAATCCTATATACGTGGTCTGATGATGACACTAGAGTCGAAGTTGTCA
GTGAATTAAGACCATTCTTGTACTAACAAGACGTGTACTAACAAAATTGCGTGTTCTTATAATTTACTGTCTGAGAGTTATGAAAACAA
TCTAAGAACTTTCTGAGCATTACTATAGAACTATTTGTATTAATAACCTGGCTCTAGGATATTTTAAGTAATCTGAACTAAACTTTATA
AGGCACGGTGTTGAGAAAACCTCAACTCATTCACATTCCTCTGAAGTTAAGAGATGAAGTCTTTCAGTCAGACTTGCACGTGTGTTTTT
TGTTCGCCCTAAACCCTCCAAGATCCTTCCTACCATAGGAGGCTCAGGTGGATTTTGTAGCATGAAGAACTTAGCCTATATGCGTGCTC

CCTTCTCTTTGAATGTACTGCATGTATTGAATAATTGCACATGATTGATTTTGGAAGATTCGAGAACCCAACAGAAAGATAACAAGGAT
GCCTATAGGCAGTTATAGGTTTGAACATTACCATTGAGGATTTTGTTGACATTGTTGTTTCATCTCATGTATGCCTATATGCTCTTGTT
TAAGTTGTTGTCTTTTTGTCTAACAATCTGCCTCCACTGTTCTATGGATTTCCGTATGGCATCTGATATTAGAACGTTATGGGATCAAG
TTTACGGAAAGGATTCAATCGATGTTACTCGGTTTCTTTTGTACGATGTATGTCTGGTTCGTTGCATGATGTGTATGAAGAAACTCAAC
TTGTTTTTCTCGTTGGTTAGGATATGAGATGGTCATTATTTTCAGTCTGAGTTTGTATTGTAAGCAGACCCACTTTTTTACATCGTGTA
CCGGCAAGTACCGACATTCTTTGAAGCTGCGTGTTCCTGCATTGTAATAGCTGACCTAACTTGCCATGTTGAATGTTTCCTCCCCCAAT
CAAGTCTTTGGTAATTGATTGGAAAAATATATAGGGCTGCTGTTCATTGTAGGCAATTGTAAAAGAGAAAAACCAAGATGGCCGCCCCC
AGTTTACGAGGAAAGGAGTTCAGACCAGCACGCACACGCACCGGGCTCCGACGGGGGAGAGAGAGAATTCTATTAGACATCAAAGGCCT
GCCGACAACTTGGGACACGACCTAATGCTGTAATCAAATTGGAGATGTTGCTACAAGAAGTTTCGGCTCTTTTGTCCGGGACCCGTTTC
TTTCTTTTTCTCCCTCCTCTCCAGTGTCTGTCTTTCTCTCTTCATTTTCCCCGGGTCCACAGCGTCTCGTCTCCCCCGTCCGCGGGATT
GATCCACGCTCTCTAGGGACGCATCTTGTGAGAGCACCGCGTTGCAGTCTTGTGTTGAGCCAAATACCTCCCTGGATCTATATACCCTA
TTCTCCAGCGCACTAGTAAGCATTCAACTGCTCTTTTCTTAACTCATGGAATAAGAGAGAGAGAATGAAAACCTGCACCAGACCTCTAT
TATAATGAAGTCCTAACTTGCAGCTTAGCTTTAATTAATTCGCACAGGGTATCATGTGCGGGCGGATTCCGCTAGGCATCGATCCTGCC
CCAGTCTATACACTTCAAAGTCTGCCGTGGCTTTACCGACGACATCATTGTATGATTAGGTGCCCAATAATCACCTTTAATCATTAGGT
ATTATTATATAGCTGAAACCGCCTAACTTAACATTATCGGTCTTCTGATTGCATTTTTGTAAAAAGAAATGCTGTCCGTGCAGCTAGCT
GTGTTTTTCTTTGTTTCTTGTTGGTTACAAAAGTTGCATGAGGAAAACGCGTGGTTTGGAAAGGGCCTTCACGATCACGCAAAAGCCGT
ATCTAGTTTGCCGAGTTGGACGAGACAAGAATGCGACAACTATCCGGACATCTCGACGGTGTAAAGATAAAGGGCCGCAGATTTGGTGC
ACAGAATCAACTTTGCGATTTGTCATTAACAAAGTTAGGCTTCTTATGTTGAAATATCCAATGGATGTGTTTAAATTTATAGGAAATGA
AATCCTTGTACCAATATACGCGCGTTTTAGGAACACCACCCCTCTCCCTTAATTCACCGGTGAAATGCATCTTGAAGTTTTCGCATTGT
CGATACCGTATTTCAACGAACATCAAATATCGTAAGGCCGTTTTGCAAAGTACTTGTCAGATCCCAGTTGTTAAACCGACGTAATGGGT
GCACGTTTGACTGTTTTCTATATAGTCAAATTTAACTACAGTTTCAGAGTAAACAGTGCTCGTAAAATAAACCATGTTTTCTCCAATGT
GTTCATTGATTAAACAGAGTTGCTGTGAACGGATTAACCCTAACTCGTAGCATGATTTACTAGGAACCATACCCATCCTGGCAAATTCC
AACACCATTTTGGAAACTGCACACATATATTTCTGACTTTGATTCTGGAGATGTGCCAACCCAATGTAAAAGACTGTCAGTTTTAGGGG
TACTGTACCCATCCTGACAGATTTGATGGGTTGAGTTCTGTACTACGTTCATGGTCAGGATCTATAGTTGTGGTAAACAATTGTCTTTA
TGTGCATGGCCTATCTTCCATAAATATAAGTTTGCTGTAGGATAAAATATTTAACTGCATAACTATTGTTCCTAACCATATTTATGGTA
AGGAGGTAAAGTTATTGCAATCAATGTTGTGTGTGCATGATCTGTCTCCCATAAATACTGGCGGTAGGATTGATTACTTTTCTGTATGT
CGAGTATTGAAGTATGATAAGCATTGTATCTGTGCTGGCATTATTCCCTAATTTGATCAATTTCTCATGTCAGTGAAAATTCTAATCAA
TTAGTCACCAAGATTCAAGTGATTGCATTGCCTATGTGCCTGGATCATGGAGCTCTCAATCTGTCTCAATACCTTAATTTTGTCCCCAT
TCCCCTACCCACATTAGTAATTACTACCGCGTTAGTGGATAAATCAAAAGTCCCAAAAAATTATATCAAAATTCACGATCAGATTAAAA
AAGCCGGTTTGTTCGGAGGGTCATCCACTGAATTGCAATGGGAGAATTATGAATGGAGCACCGGTTCTCAAGCATGTATCTGAATTCTC
AATCAGTACGTGATAATGATGAATCACGGAATGTAAGATCCAGACGCGCTCGAACGGAAACAGGTTCTTTTTGGAGGCGGGAAGGTGAA
TTGAAAATTGCTGTATAATTATGCTCCTCACGCGATGAGCGATATTAAAAAGTGACATCAGCGAAGCACGCTGCGCAAGGAAAATTATG
ACTAGAGAAAGTTCACCTTTGGGTTGATTTTTTTTTTTAGTTTTTCCTTCTGTCTTTTTTTTTTTTTCATCCTGTAGACCTACATGGGTAT
ATTTGTCAACCATTTATAACCCCACTCCTGTGGAAATGATGGAGGGGTTGGAGTATCTGGGCATATTTAGGATCTGAAGTATCTGTAGT
GTGGTCAAGAGTCCGAGATGAGAGATAGCCAACCGGTCCCCCGGTTTCCCCCCATACGATACACCGCGTGAATACTCAATGAGACACGG
GCAGAACAACGAGGAGTTGTCAACGGACCGAGCAGAGCGCGCATTCGTCCCGGGGAGGGACGCGCCGTCGCACGGCGGGGCTGGCTCGC
TGCCGGCGCGAGTCACACCGGTAATTTGTTAGACGACACGAGGGGGGACCATCGCGAGAAGTCATCGGGAACACGATGAATACATTCAT
GAAGTACTTCCAACGGTCATTTATCATATCAACGCAGGAACGTGGGCTCTTGTGCTCTGTTGCTCAGCTGAGATTCTCTTTTTTATCAT
GTTCACATCTCCAGTTCAGTGAAAAATCACCGAAATAAGGTCATGTATAGTGAATCCTTTACACTGCTATATGTTAGCATTTTAGTACG
GCACCAAGCTAAGGCTGTAACCGTTTTTGAGGTTTTTCTTTAGTAACGTTTCTCAAAAGCTAATTCAGAATCAGTTTGTTTGTGCAGAC
TTCATGTTTGTCCAAATACCGCTTAACGTTAAATAAGTTCGTCCCGGTACTTCAAAAGAAAAAAAAGCGTTTAACATAATTACGGCTGA
AGGGGTCGACCCTGTGTTAGAACCCAAGTTGTTTGCATGGGTAAAACCCCCACTTGATATAGAGGTAGACTGCTTTCGGAATTCATAAC
CCTTAGGCTATAGCAAGACTTGTACTTTTTGTACGAGCGTTTGGATAAGAAAACAGAAAACAGCACCGGCTTCCAACCGCAGACACCGA
CCGGTTTGTTCGCTCCGGCCGGAGCAACGAACGCCGACCAAATTAGTTTTGGGAGACCATAGACGAAGGGGGAAAATAGCTAGCTGGAC
AAGACTCTTATTGGGATGTGAAGGGTGCTGAGCGGTGTCTTCTATGATGGGATTTGGTTGACTTTAGTCAAGTTGTATTGTCCCCCCTG
AAAGGGGTAAAAATCAACTTCGGAGACCTGGGGACCTGGGATGATGTCTTGATCGTGTTAAGGGCGCTGAAGTGGCATTCTTTTGTCGG
GCCGTGGAGTGGGAGATGAGGGGATCAGTGTAAAGATTGCCTGTTGTTGTTGGTTTTTTGTTTGTTTGGTGGATGGAGAGAACAACGAT
GATGCGATCATTATTGGGGCCGAGCTTGGTGGGTTAAATGCGCCGGGAGCCCGGGGATGAAGATGACTGTAACCCGCTAACCAATCACC
ATCATCATCATGATCATCATCACGATGATGATGATCATCATCATCATCACAACCGTATCGTCACCATGATGGCCGCAACCAACACATTG
CAAAACACCCCTGTATCCACACCACTACTCCAATAACTTCCGATACAAACACAATGCTCATTTCACACTACAAGTGCAGAATCGTCCAG
ATCACCATATAGAATCATCATCATAATCACCATATAGAATCATCATCATAATCACCACCAATCGCCACCATCACACCACATCACCACCA
AAATCACCATTACTACCACAGTCATCACCACTGCCATCCTCAAATCTTCACATCATCAATAAAATCGTCATAATCATACTCACCACTGT
CTACCTCATCGTTGTCACCGTAAAGACTATGTCCAAATTAAAATCGAAACCGAGGCTCTCATTGCTCTCATTTTTTATTGTTTCATCCT
CACTACAAATAGAAAACTGGTGCCCTCGGTTTCGTTCCTAACATAGAATTCGGCTTTAGAATGGGAAAACCTTTTCATTTAAAAAACGA
ACAATCACATTAAATTTTCAAGAATTGAAGAATACAAACAACAACCTATACGATTGTGTGAACAGTGCCGGACCATTTGGGATTGGGTG
CAGTGTATGTTTATCTTTCATGGTCATTAATTAAAGTTAGACGACGTTAAGTGTTGTTGTGACCTATGTACCCTTGTAGAATAAATTAT
GAAACGGACCGCTTGGGGAGAACATAGACTTCGGCTCATTAAACGTATTCAAGTTGGTATCTTCTGGACATTTTACGTGCTTTTGGTCA
CGTATAATCATGATAATCACAATGCCCTCAGATCTTGTGAAATAGAAAAACCATTCAATTCATTTTCGATATTCAATTGAACGGTATAG
GAGTGCCAACAAGCGATTTCAGTAAAGATAAAATGAAACGAAGTTGAGTACGAAATATAAAAACACCATAAAGTCCATTTCTGAAATTC
ACAGCAGAATAATATTTTGAAGAGTTTAAAGTATGATGAACAGATTTTTTGAGAATAAGTTGGAATTGAGTTTGAGTTTTAATATCTCC
AAGACGAAACTGATCTTTTGGGGGACTTCTTCGTGTTTGTCATGCTAGTTTTAAGGCAACACTCTTGCATATATGTTGAGGAATTTGGC
GTCTTAATGTTGAAACTTTTCTGTTAAACAGAAAGACACAAATATATCTGCAATGTGACCAAGGAAAAATCTTGTGGGAAATAGAGCAT
ATTAGTTTCCAGAACAATAACGAGGGAGTTAAAAACTGCATAATCGGAATACTTTGAAACTAAACAAAAGGATTATAGGAATGCAGTAT
CAGTTGTATAATGTTATGGTGAATATTGAGGGTACCCTTTTCCATATACCAGCATCATCGCTATATACTGTTGACATTCTTCAACTTAA
AAAAATAAGATGAGTGTTTATCCCGTTCTATATCATACATTACATATTTTGTCTTCTTTTTCAATGAATCACCCAATATGCCTTAAAAC
GAATACAATCGTCTCAAACAAAGCGTCCGATTTTGTACTATGCGATGGAATTACTCGCCTTCCTGAAACGAAAAATCAATCACTGGGGA
ACGTTTAATAGTGGGTTGTTTTTTTTTGCTTTTGAGCTAAGAGAATCGGGACGCTCTGTGTATGTGGACACGAGTTATCATTATTGTGTG
CTTGTTGATTCGACATGAATGCCATTTATCATCTTAATTACTCGTGCTAGCATTTAGTCGTTATTCTGCCTTACCGCGTGGCCCGTATG
TCATGCAAGCGAAACGGCCCAAACGATCTGCCCTGCTATCGGTATAACTTTCACTTTTCAAGACCGAAAGTGAAATTTGGGAGTTTTGA
AAGTCAATTCTAAACCAGTGTGCACATGTAAACCGCTGTTGAAATTGGGCGGGTAAACAGCTCATTTCCATATAGAACCGCCTGCTCTA
TTTGGATAAGAATGTTTAACAACTCGCCACTCTGTTTGCGTTGTAAATGTGGGCCGTGTGTGTAAAAAAAGTACAGTCTTTAAAGTTGT

CTAAGATATGCAAATCGGCAAATTTATTGCTGGAATTCCTTTCTTGTTCGTCGTATTAATTAACTGGTGTCGTGCAGACTGACAAAGGC
AATTTTCATCATTAAAACATCAAGAACGGATTTTTCTCTTCGTTGGGCGCCATATTTTAACTCCTCCCCGATCGATCGAGCGTAATAAA
CGATGGCCCTAACTTTCCCCGCGTATATTTTTCTTAGATTCGTTATGCAAATTAGTGGACTAAATTATTGCCCTTTCCATGTTTAGACA
ACCGTTTAAATGACTCTGGCCACACAATGGACACGTGTTGAAAACATGTATGTGCTTATTTTCTAGCTCCGCGACACCCCCTTTTTATG
CTGGGAACGGAAAACAAATACAAATTTTACCAAGTCAACACATCCGTGCAAATCCCGATCGTCATAGGCCTATGGTTTGCATTACCCAC
CCCTAAAACAGGTCCCGATAAAGAGGAGGAAAATATGAAATTAAACAGAATAAAATAAAAATGCCGCTGCAGCCGGCAGACCCAGTTTA
TCAACCGGTCGATTGGAACTTTTATTTCATTTTCCTTTTTCATTTTTCCATGTTCACAGCTGTTCATTTTCCGTGCATGTGCATAGATT
ACTACTGAAATTCGATCAATGTTTTTATTAATTGGTTGTTTAAGTTGGGAGTTTTTAAGCTGAAGTTTTTTTTCCCCTGGCATGTTGAA
CTCCCACCACGCTACACAACTTTTTGCGAGTTTTTTTTTCAAAAGCGTTTTCCTCGCAGCGTGCAATGCACTGGCAAAAAGTGCAAGTT
TATCTCCGACACGCGCCGCGAGCCGCCGGTGAAGGTTGGGTCTGCTGGCGTCGATGGGGGCGCTATTGATAGTCGCAGTGTATGACTCG
CTCTCCCCCCAAAAGTTGCAAAGTCCTGGGATCTAAGACAATATCAGCATGTTTTGTTGTCGAGTCCATCACAAGTTCGCCACAACTCC
AGTCAGAAGACAATCGCAACTCCACTTACAAGTCCAGTCACAAGCAATACTGCATGAACAGTTACAAAGAACTTCTTTTGTCGCAGGTC
ATTTGGAGATGGCTTGTGACTCGACTTGAGGCTTGAAATGGACTTTTGATTTACTTGTGATGGACTTTTGATTTGCTTGTGATTGACTT
GCGATTGACTTGCGATTGACTTGCGATGCACTTGTGATTGACATGTGATGGACTTGTGTGAGGGGCTTGCAATGGACTTGTGATGGACT
TGTGCTTTGCGATGGACTTGTGATGGACTTGTGGTGGACTTGTAATGGACTTATGTGAGGGGCTTGCGATGGACTTGTGCTTTACTTGC
GCTTTAGTTGTGATTGACTTGTGATGGACTTGCGATGGACTTGCGATGGACTTGTGATGGACTTGTGTGAGGGGCTTGCGATGGACTTG
TGCTTTACTTGTGCTTTAGTTGTGATTGACTTGTGATGGACTTGTGATGGACTTGCGATGTACTTGTGATGGACTTGTGATAAATTTGT
GAGGGCCTTGCGATGGACTTGTGATGAACTTGCAATTGATTCAATAATCAGTTCTCCATGCTAGATAGAACTGAGAAACCATGATAACC
AACAGAGAAAGCACGCAGATTAACCAGAAATGTGAAACACAGATGCTAAAATATAAAAGAAAAAATAGATACCTGATTGAAACTGATTG
AAAAAAGAAGATATTTTTAGCCCCTGCTCGAACCGGACACTTCATTTCCAATTATGCAAATAACTCCTCACTTCTCTCCCTCTTCGTCA
CGGTAGGACCAGGCGCCTTCCCTCCCGACCACCTTATCTGATAAAACAAGCGGAAAATTGATGTCATTTGCTCGCTGTTTTGTAGCCAT
AATCGCGCGTCTTAAAAGCTGGCCATGGGCGTGCATAATATGGCAGGGGGCAATTTGCAGCATCTCTTGAGATACCGAGGGGTTTTCAT
CCAAGGGAGTGATTAAGAGAAAAGAGACCGCGGGATCTAGGCTCACAGACCGGCCAGTCGATCTGATTGTCTCGTGATATGCCGCCTAA
TATAAATACTTCTCAATCCGTAACACTGCTTTTTTGTTTTTAACATCCTTTCATTTAAGATGCCCTGCCTGTTAATGTTAAACCCTTTC
CCTGTAAGGCATCCACTAACCTCCTTAAGAATATCACCTTCGCCCCTGCTAGACAAATGACAGGATGATTTTGTATTAACTTGCCTTCG
CACAAAAGCACTTCACCCTTACACACTGGGTTTGACGCTCCCAACAAATAGACCATCTGTAAACGCGGTAACCCCTCTCAGACCCCACA
TCAAGCCTCTCACCATATAGACAAATTACTTGATGATAGTCTGCTCTCAGTTTTTTTGAAAACTGGGAAATCGATCCTTCTCTCATCTT
TATATAAAAGCAGGGGTGAATGTGATGCTAAGTTTCCCCAACGCCTGTTTAACAGTCGGCATCTTAAGAGCAACTCATTCGGTGACGGC
GCTCGGCCACCGCGGGCCGGTTGGCTCCTGGTCGCGTGTCTCCAAGTGCCTGCCGTGGAACGCTCCACCGTTAAACAGTCACGACTATC
GCAATCTCGGACGAAAACAAAAGAAAGAAGACATCTCGGCTCGATCAAACCCATAATCTTCATTGCAATAATTACTCGCTACGGTACTC
TTCATCATCTTGCGGCAGTACAAAAAGTGTCCATGCTGCCTGTCTGGATGATACTACAGAAAAAACTGATTAAAAAGTCTTTCAAGTAA
TGACTGCTTGTCGGAGTAGATTAGTCTGGGCAATCTCCCTAATTTCGGTTGTTTGGGGGCCGGGGTCGGCTGTGTATCGGGGAGTACTT
TTTGCAGTTGCCCCTCTTGGATGAGAGGCCCTCTCTACGTTGTCGCTTCCAATCACTGCCACAGACTTCAGACTGGCGACTTGTGTCCC
GAGACCTAGACTTGTGTCGCTACGAGGGAGAGGGAGAGAAGTACCAACAAATAATTAACCAACAAAAATATCAATCTCGGTGGTGTTTT
GAGAGAGAGAGAGAGAGAGGGGAGGAGAGGTGTACTTTTGTTCCCATTGCCCAGTTTGTAATTTACACTCTAGCTAGGCTGATCATGTA
GCGATTATACACGGGTCTCTCAAGTGCGTGAACATGGGGTTGGGGAAGGGGAAAACATGGGCACTTGCTAGAAATGGCGCCCAGCCTGT
CGACATTATTGAGCCTTTTTTCTGTGGATGGTTTTTTTTTCATCATTGAGGTTTATGGCTTGACAATGACATGATATCACTAGCCGGAT
ATGAGACTCGCGAGTGATATTTTCCGTAGGAAATCAAATATCGGTGATTAAAACAGAGCTCATTAATCCCAAACTGTCAAATTAACACT
TCATGTGCAGTGCTTGGGAGTATCTTTCTGTAGGTTATTAATTCCTGTCTTTTTTTATTGCATCTTTTTTATGGCTGTTACAGACTACC
TTAATTTTCATTACGTCTTATTTATCCTTTCATGAACTTCAAGTAACCCTTATCAAAGGTCATATTTAACATATTTCACATATTTCTGA
ATCAGCCAATAATCGTTGAGGTAAGATAACCCCTTTTGCCAGGTTGTGAATGTTCTGATTTCTTATTGGTCACCAGG

**>*PmLox* exon2**

GTGGGCAGGAGGGCAGCGATTGGCTAGCATGCAACAACGTCACGACGCGGACGGCAGGTGCCTCGTTTC

**>PmLox exon2**

GWAGGQRLASMQQRHDADGRCLV

**>*PmLox* intron2**

AGGTCTGGTTTCACTTTTTTGTTTGTTTATTTTTTCCTTTTACTTTCACTTTCTTGCGAATTTAGCAAAATCTTAACAATAACAAGGGC
ATTTCTTGCATAAATACTACTAGTGTTGGCTAGAAGAACAAAGAAAAGAAATCGTGTACAACGTTTATGGGATTTTGGCTAGAAGAAGA
AAAAGAAGAAAAACAAACTAATCAGAACAAAAACAATGCTTTACCAGATCTTGTCATGAACAACTTAAAACAAAACATGTACCTCTATG
ACCCGCAATAGCAGTGTGATATATAAAACAATCCCTTAAGCTTGATCTTTCGCCCGATGCCTCGGTAGCCAGGTCCTCAAGACGTGAAG
GAGGGTGGTTGGCCGGTGAAATTGATGAGATCTTTCGGGGGGCCGATCCGTGAAAAAAAAGACGCACTCGTGGTTGCGAGCTAGCTGCG
GAATTCAGCCACAGATTAGCGAGTTGAGCAATTGCTACCGGCATACGGAGGGGGATGGCGGCGCGGATACAACAGATTTGGTTCGAAAT
TGAGTGGCAGAATGTCTTTTTTTTCCTTTTACACTCTCCACTCTGTCTTTCTCTCTCTCTCGTCCTCTCCTCAAGAAAGTGAGAGAAGT
GTTTTCGGGGGACTTTGGCACTGAATAATATCACTTGCGTATCAAGAGGTGGTAAATTTGCTGCGAAGAAGATGATATCCCGACGGACA
CGTTACAGAGTCGTGGCGATTTACCCATCCATTTATTCCACAACATTTATAATTTCTGGACAGGAATGAAAAATACAGAATATTTTGAA
AACAGAGTGAAGAAGGGTCCATTTTAAAATACGAACACAACTGAACCGTGCACATGAGAATGTTTTGAGCCCTCCCTTGCATAAGCCAC
ACATCATATTTTAGACGGAAACCAAAATGTAGTATACATTGATTGTACTGCAGTACTGTTTGAGGTGTGTGCTCTTGTCCAACAGGGAT
ACGCATGAGTGTAGGCCTCCCGTGCATTCATTGTTAATGAATGAACATAACAAAACACATCCCAGAGATGGCTGTCGAACGCAATCCAT
CTATAGGCCAACTTTGGAGTCTGGTTGGACAGCCTATTTATAGCTTCTATAAAGACCATTATCATGTTGCGGCCATCCTGATTTTTCCC
CATTCAAATCAACGTAACCAAAGCGAGGCTGGAAGGAAATATAGTCTGGTTCCTTTTTGCACAAAAACCTTATCATGCAGTACTGTTAT
TTTGATACAGGGAACATGACTGAAATGGTGGCAGCGTGATAAAGGTCTATAGCATCTTTTGTTCGAACCTTGACCAGTTTCCGGACACG
TTGGGACATCACCATGTGCGAGTTAATTTGTTAACTGCTCCATTAGCATAATTTGTTTAATGGCCCCGCGGTTCAATGTGCTGGTGTGA
ATAAATGCCTGGCTGTAAATTGATTTTCGATTTGTCACAAAGCAGCCGTCGAACGCCTGGCGACAAGTATTTTCTCATTCATTGGAATC
GGCCGGTCGGTCAGTCCATGTTTAATAATTATTAGATTATACATGGAACAAAGAAATCGACTGGAGCGGGGTTTGAACCTGCGAGTTCC
AGAATACCCTTCCGTTGCTTTACCAACTGAGCTAATGTTGGCTGGTTCCCCAGGGTTTTTTTCAAGGGACGCTAGTCAGAAGCCATAAA
CCCGTATAATACCATGTAGCCAAGGATCATGCTCAAAGTCACGATGCTAGCAACATTAGCAATGTTGCAGACCTGGTAAATGACATTTG
AAAGCCAAAAAGCTCTGGCTTGACTTTCACTTTTCATGTTTCGCTAAGTGGAACAAAATGGCTCACCAGCTGTATCACTGACTTCGCTT
GTGGGCCATAAACACCCTAAAAGCCCGCTGAAGTACCGTTAGCTCAAACAAGCAAACAGGTTTTTTTATGGTGTAGTTGCAAATTTGTC
CAGCATTCCCCAGAAGCGAGTGTAGATTGAGCTGGTTTTTGCCGTCAGACATCCAAAAAACACTCTTGCCTACACCCAGCCATTTGTTT
AACAGCAGACTTGTCGGGCCCGCCTAATCGTATTCCCGATTCCCCTAAGAGCCAGCCACTCCAGATTATGTAGACCACAGAGCCACCTT
ACCCATTACCTACCCCATATGTAGGCCACGGAATAATTACCCAGCCAGAAAAAAATGATGGAGAGTTATAGACAGATAGCACATTATTC

GCCGGATGGCCGTTATTATACCGTTCTTTCCTAATTCCGAAGGCACGACCATCAGAAACATTATCGCCGGGCGTCGTAACCAGGGAACC
CATGAAAGGCACCTCACAGCTCACCCCAAAAGCCCAGCCGGGCCGAATTGGAATGTCATATTTCGTGTAATAGGGAGTTGTTCCGCTTT
ATATTTATGTGCTTTGATGACACATTATAATCGAGGCTTTATATTTACTGGACCAGGCAGAGAGGAAGGGGGAATCGTGGCTTGCTTGG
CAGGCCAGGAATTGTCACTTTGGAAATCGGTTGTCCAAAAAAAGACGACCTGAGTAAATGAGGAACGTCTGTCCTGTCCCGCGCTAGGG
ACGGGAAAGGAGATGTGTGGTCTACCCATTAGAAATAATTAGTGATAATGGCCCGTTGGCTATGGCAGCGTGTTACGTATTAAATGGAA
CAACTTGTTACACTGACCCAGAAAAAAAGAGACAAGCTAGGATGCTCTTTGAATCCTCGGACTGGCAATATTCAACTACAGTCAGTGTG
GCTAGCTCTGCGGCACGTTATGTCCATTTTTCATGGAGAAAATTGAAATAAAGACAAGAAAACAACAATTTTGCATCTGGAGATACTGA
GTCTTCAGTCACACAAAAGGACAAGTATCAACTGACGGCTTTATAGTTGAGCATCGTTATTGAAAATCATAATCCACAGAATTGTGAAG
TAAGCAGATGAAATAAATTCAAGGAACCAGAAATGAACACTACTTGAGCTTAGTTTTGAATAATAAATGTAAATGTTTTTAAAGCTAAA
TCTATCAACTCATCGTTTTGGTTTGTTAATTTATTAATTTGTTTTATTTAATTTTTGCTTTGGATCAGTCAGTCCGTCCTTTTGTCAGG
CTTTTGTCATAAGCTTAGGTCTGGTTTCACTTTTTTGTTTGTTTATTTTTTCCTTTTACTTTCACTTTCTTGCGAATTTAGCAAAATCT
TAACAATAACAAGGGCATTTCTTGCATAAATACTACTAGTGTTGGCTAGAAGAACAAAGAAAAGAAATCGTGTACAACGTTTATGGGAT
TTTGGCTAGAAGAAGAAAAAGAAGAAAAACAAACTAATCAGAACAAAAACAATGCTTTACCAGATCTTGTCATGAACAACTTAAAACAA
AACATGTACCTCTATGACCCGCAATAGCAGTGTGATATATAAAACAATCCCTTAAGCTTGATCTTTCGCCCGATGCCTCGGTAGCCAGG
TCCTCAAGACGTGAAGGAGGGTGGTTGGCCGGTGAAATTGATGAGATCTTTCGGGGGGCCGATCCGTGAAAAAAAAGACGCACTCGTGG
TTGCGAGCTAGCTGCGGAATTCAGCCACAGATTAGCGAGTTGAGCAATTGCTACCGGCATACGGAGGGGGATGGCGGCGCGGATACAAC
AGATTTGGTTCGAAATTGAGTGGCAGAATGTCTTTTTTTTCCTTTTACACTCTCCACTCTGTCTTTCTCTCTCTCTCGTCCTCTCCTCA
AGAAAGTGAGAGAAGTGTTTTCGGGGGACTTTGGCACTGAATAATATCACTTGCGTATCAAGAGGTGGTAAATTTGCTGCGAAGAAGAT
GATATCCCGACGGACACGTTACAGAGTCGTGGCGATTTACCCATCCATTTATTCCACAACATTTATAATTTCTGGACAGGAATGAAAAA
TACAGAATATTTTGAAAACAGAGTGAAGAAGGGTCCATTTTAAAATACGAACACAACTGAACCGTGCACATGAGAATGTTTTGAGCCCT
CCCTTGCATAAGCCACACATCATATTTTAGACGGAAACCAAAATGTAGTATACATTGATTGTACTGCAGTACTGTTTGAGGTGTGTGCT
CTTGTCCAACAGGGATACGCATGAGTGTAGGCCTCCCGTGCATTCATTGTTAATGAATGAACATAACAAAACACATCCCAGAGATGGCT
GTCGAACGCAATCCATCTATAGGCCAACTTTGGAGTCTGGTTGGACAGCCTATTTATAGCTTCTATAAAGACCATTATCATGTTGCGGC
CATCCTGATTTTTCCCCATTCAAATCAACGTAACCAAAGCGAGGCTGGAAGGAAATATAGTCTGGTTCCTTTTTGCACAAAAACCTTAT
CATGCAGTACTGTTATTTTGATACAGGGAACATGACTGAAATGGTGGCAGCGTGATAAAGGTCTATAGCATCTTTTGTTCGAACCTTGA
CCAGTTTCCGGACACGTTGGGACATCACCATGTGCGAGTTAATTTGTTAACTGCTCCATTAGCATAATTTGTTTAATGGCCCCGCGGTT
CAATGTGCTGGTGTGAATAAATGCCTGGCTGTAAATTGATTTTCGATTTGTCACAAAGCAGCCGTCGAACGCCTGGCGACAAGTATTTT
CTCATTCATTGGAATCGGCCGGTCGGTCAGTCCATGTTTAATAATTATTAGATTATACATGGAACAAAGAAATCGACTGGAGCGGGGTT
TGAACCTGCGAGTTCCAGAATACCCTTCCGTTGCTTTACCAACTGAGCTAATGTTGGCTGGTTCCCCAGGGTTTTTTTCAAGGGACGCT
AGTCAGAAGCCATAAACCCGTATAATACCATGTAGCCAAGGATCATGCTCAAAGTCACGATGCTAGCAACATTAGCAATGTTGCAGACC
TGGTAAATGACATTTGAAAGCCAAAAAGCTCTGGCTTGACTTTCACTTTTCATGTTTCGCTAAGTGGAACAAAATGGCTCACCAGCTGT
ATCACTGACTTCGCTTGTGGGCCATAAACACCCTAAAAGCCCGCTGAAGTACCGTTAGCTCAAACAAGCAAACAGGTTTTTTTATGGTG
TAGTTGCAAATTTGTCCAGCATTCCCCAGAAGCGAGTGTAGATTGAGCTGGTTTTTGCCGTCAGACATCCAAAAAACACTCTTGCCTAC
ACCCAGCCATTTGTTTAACAGCAGACTTGTCGGGCCCGCCTAATCGTATTCCCGATTCCCCTAAGAGCCAGCCACTCCAGATTATGTAG
ACCACAGAGCCACCTTACCCATTACCTACCCCATATGTAGGCCACGGAATAATTACCCAGCCAGAAAAAAAATGATGGAGAGTTATAGAC
AGATAGCACATTATTCGCCGGATGGCCGTTATTATACCGTTCTTTCCTAATTCCGAAGGCACGACCATCAGAAACATTATCGCCGGGCG
TCGTAACCAGGGAACCCATGAAAGGCACCTCACAGCTCACCCCAAAAGCCCAGCCGGGCCGAATTGGAATGTCATATTTCGTGTAATAG
GGAGTTGTTCCGCTTTATATTTATGTGCTTTGATGACACATTATAATCGAGGCTTTATATTTACTGGACCAGGCAGAGAGGAAGGGGGA
ATCGTGGCTTGCTTGGCAGGCCAGGAATTGTCACTTTGGAAATCGGTTGTCCAAAAAAAGACGACCTGAGTAAATGAGGAACGTCTGTC
CTGTCCCGCGCTAGGGACGGGAAAGGAGATGTGTGGTCTACCCATTAGAAATAATTAGTGATAATGGCCCGTTGGCTATGGCAGCGTGT
TACGTATTAAATGGAACAACTTGTTACACTGACCCAGAAAAAAAGAGACAAGCTAGGATGCTCTTTGAATCCTCGGACTGGCAATATTC
AACTACAGTCAGTGTGGCTAGCTCTGCGGCACGTTATGTCCATTTTTCATGGAGAAAATTGAAATAAAGACAAGAAAACAACAATTTTG
CATCTGGAGATACTGAGTCTTCAGTCACACAAAAGGACAAGTATCAACTGACGGCTTTATAGTTGAGCATCGTTATTGAAAATCATAAT
CCACAGAATTGTGAAGTAAGCAGATGAAATAAATTCAAGGAACCAGAAATGAACACTACTTGAGCTTAGTTTTGAATAATAAATGTAAA
TGTTTTTAAAGCTAAATCTATCAACTCATCGTTTTGGTTTGTTAATTTATTAATTTGTTTTATTTAATTTTTGCTTTGGATCAGTCAGT
CCGTCCTTTTGTCAGGCTTTTGTCATAAGCTT
**>*PmLox* exon3**
AGGTGCCTCCTTTGTAGACTTTGACGAGAACAAGCGCACGCGGACGGCCTACACCCGAGGCCAGCTCCTAGAACTGGAGAAGGAGTTCC
ACTTCAATAAGTACATCTCCCGGCCTCGTCGGATCGAGCTGGCCGCCATGCTGAACCTGACCGAGCGACACATCAAGATCTGGTTCCAG
AACCGCCGCATGAAGTGGAAGAAAGAGGAGGCCAAGCGGCGGCCCAGCGGCGGCAAATCCGACAAGGAGGAAAGCGAGGACCTCAAGCC
CGACCTCAAGTCAAACGGGGACTCGTCCTCGGACAAAGAGGAACTCTCATCGGAAAAGAGCCCTCTGTCGCCCGGCAGCGACCAGGCGC
TGCAGGGGGTCAAGGGGGTGGACGCCTTCTCGCCCTCTACGTCACCGCGGAATCTGAACATGACGGCTGCGCACCCAGCAGAACATCTC
ATCGTCAAGTCTTTCTGA
>**PmLox exon3**
SGASFVDFDENKRTRTAYTRGQLLELEKEFHFNKYISRPRRIELAAMLNLTERHIKIWFQNRRMKWKKKGGQAAAQRRQIRQGGKRGPQ
ARPQVKRGLVLGQKGTLVGKKPSVARQRPGAAGGQGGWTPFSPSTSPRKSEHERLRTQQNISSSSLSDNERGLLLKVRKFDKNVETFCG
TGAAAFAI

## PmGsx:

**>*PmGsx* exon1**
ATGTCTCGGTCTTTTTACGTGGACTCGCTGATTTTAAACCAGCCCCCGTCTAGCGAGAGGGAGCACCCACGCACCCCTCCCTCCATGCA
CCTCCGCACTAACGGTGAGCCCAAGAGTCTGAGATGCTACCAGACCCATCACACGGACACCATACCCACCTGTCCCATCTGCGTACGGG
ACCAGAGCCAGGCGACCTGCGTGGGCCGTCTGCCCGGGATGCCTCCCCCGACCTCCGCGGCGGTGGCCGTCTTCAAGTCCCCGCTCGCC
AGCCTAGCACCGTCGCATCACTCGCCCAGCGAGCATCACCGCATGCTCCCCACCAGAAGTTACCTCGAGTCCTGCCGGGACCATCACCT
TCACCCGTCAAGTCTGGGGCTTCCCCCTCCCCATAGTATGAGCCAGCACACGCCGCCATCCTCCCACACAAGCTACCGCCCCACCATAG
ACCCGAGGAGACTCCAATACCTACCCATGG
>**PmGsx exon1**
MSRSFYVDSLILNQPPSSEREHPRTPPSMHLRTNGEPKSLRCYQTHHTDTIPTCPICVRDQSQATCVGRLPGMPPPTSAAVAVFKSPLA
SLAPSHHSPSEHHRMLPTRSYLESCRDHHLHPSSLGLPPPHSMSQHTPPSSHTSYRPTIDPRRLQYLPM

**>*PmGsx* intron1**
GTAAGTCGAAGAATTTTTATCAATATTTTTCTTGTTTTGATTTCATTTTTTTAAATTACAGTTTAGCGTCTGACACCTGGAGTTTTCAT
CTAGTTTTTCTTAAAAGATAATGTCATTTTAGACTAACAAAAAAACATTCAATGTTATTTTTTTGAATAACGAAAAAAAAATGACCAAT
ACGTTGATCATGGGCTTTATTCAAAACAAAGCTAAGCAAGTGCGAAACTTTCATTTCTTTTCCATTACTAAAATTTTCAAATTAATTAA
GTTAATTTCATAAGAAAACTGTTTTCTTTTTATTAAGAGTTAGTTTAAACTGATTTACACCAAAAGTATGCCGACTCCCATGTTCTTTC
TTGTTTTATCCTTTGTTTTCGGTGCTTATTGTTCTACAGACACGTAACTCTGAGCGCGCCAACGATCACTTGGTCAGTATCGATTCCTC
GTAATCGAGAAATTCGATACCAAACGGCTTCCATCAAAAGTCTTTATCTTTACGAGATGTGACACTAAGCGTACTCGATCCCCGTTAAA
AAAAAAACCCTTTGTATCCATATTATACTCGATAAAAAAAAGAGATTGAAAATCGATCGGAAGTGATGTGTTGCCGTTCTGTCATTCTC
ACTTTGGATTTTGGAGAAAGAACAAAAATCAGTGCATATTTTGGACGTGGGAAATGACAGATGATTTCAGCACGGGTGATCGGTATGCG
CCCAGATGTAGTATTCCCAGCGTTGGGGTAGGGTGATGTCATGGGTTATCGACACAGGACAGAATTAGTACTGTTACTTTATCATCAGT
CGAATATTGCGAAATAGGCAAGATAATATTTAAAAAACCGAAATACTGGACTTTAATCTACGTGCGATTTTGCCCCTCATTTGTGCTTA
ATCTTATTTAGAGATAACAGCGTCAGATATTTGCGTGGAAATGTTGACGCTGGTGAGCTCACATTCACAAACACCAATTATCGATACAA
AGCTCTTTTGTGTACTTTCTCTTGGATTGGGTGTGAAAATGGACCCTAAATACGGAACAGATAACCTTTGCACAGACTGCTCCATTTTT
AAATATTCTCTTGAACGCCATAATCACAAAAAATGCATTGTTTAAAGGGCAAGGGCAAATTCATTGAGTATTTTTGGACCAATTATGGG
CTTATCGGCTATCAGCTTATTGGCTGTCAGGCGCGTAGGCTCACACGCACGGGATCATCACTCGAGTATACCTTGGATTTTTTGTTTCT
GACTGTAAAACTAGGATCGAATTTCCCAAGAATAATGTCCAAATTAAGTAAATTTTAAGCCCTGCCTGACTGAGAAAGATTGGTCCTGG
ATCAGACTTTTCTTTTCCGACGTAGGTTTCTTAAAAGTGGAAAAGTTAGGCATCAATAAACTACCATTGTTCTAAACAAACAAACAAAC
AAACAAACAAAAAACAAAAGAAGGAAAAAACACCGAAAATTGCAAAGTAAATTAATTAAGATTTTCACGCTTGAATATTGCGATTCAAA
AGACTTTTGGGTGATTGCCAACGGCAAATGCGTTTGTTTACTGCATTAGCATTAAGAACGGTCGCTTAGGGAGTGGGAAATGAACCATT
GTCAATCGAAGTCGACTCCGTTTTTTGCCATAAATACCTTTGGATGACCAGTTACGCCACTCCTGTTTAAAAATGGAATACGACTTTGC
ATCCAAATATAGTATACTAAACGATGACGCCACGCGAAGCGTTACACTTACAATGTTCAATGTGACCAGCTAACTTTAAACTATTTTTA
CATGATTTGATCTTCACTGCAGTTTTAGAAACTTGTCTTCATCTCCACTGATAGCTGTGACATTTTCTAAAGCACGAGATGTTTTCATC
ATGTTTTGCTTTTTTTTTTCTCTTCGACTTTCTACTCCAACCGGCAATAAAATGGACCTAAATGGTTGAAACAGATTTAGAATACACAA
TTAAGACTATGATGCGGGCTTTCTTCTGAAAAAAATTCTGGACATTTCGAGATTGTTCCACGCATTCTTTCCCCAAAGTGATTGTATTT
TGTATTTTTTTCAATCAGCTCTGAACCATTCGGGAACGAAACTGATCGAGTGCGTGACCGCCATGTTGTTTTTTCTTGAATAGCTTTTG
ACCATTCGGGAATGGACCAAGCGTATTTCAAACTGATCACGTGCGTGACCATCATTTGTTTTGTTTTCTTGTAG
**>*PmGsx* exon2**
CTCTTGACCATTCGGGAATGGACCAGGACCACCTGTCCAGCAGCAAGCGCATCCGCACCGCCTTCACCAGCACGCAGCTCCTGGAGCTA
GAGCGAGAGTTCGCCTCCAACATGTACCTGTCGCGTCTCCGCCGCATCGAGATCGCCACCTACCTGAACCTGAGCGAGAAACAGGTCAA
GATCTGGTTCCAGAACCGGCGGGTCAAGTACAAGAAGGAGACCAAGGTCCAGTCACGTGACTGCAAGTGCCAGCGGATGACGTCATCGC
GGGCCAGCAACAGCAACGGCCGGGCTCGCTCTTCCTCGCCCGGCAGTTCTGCGCGTGCGCCCATCGACGCCGAAGACAAGCGGCAGCCG
GAGAGAGATTGA
>**PmGsx exon2**
LDHSGMDQDHLSSSKRIRTAFTSTQLLELEREFASNMYLSRLRRIEIATYLNLSEKQVKIWFQNRRVKYKKETKVQSRDCKCQRMTSSR
ASNSNGRARSSSPGSSARAPIDAEDKRQPERD

**Fig. 2**. Phylogenetic analysis of *Patiria miniata* ParaHox genes together with all ANTP genes from the sea urchin *Strongylocentrotus purpuratus* (Sp), the protostome *Drosophila melanogaster* and the cnidarian *Nematostella vectensis* (Nv), rooted on *Lim*. For methods, data set and abbreviations, see [Arnone MI, Rizzo F, Annunziata R, Cameron RA, Peterson KJ, Martinez P. Genetic organization and embryonic expression of the ParaHox genes in the sea urchin *S. purpuratus*: Insights into the relationship between clustering and colinearity. *Dev. Biol.* (2006) 300, 63-73].

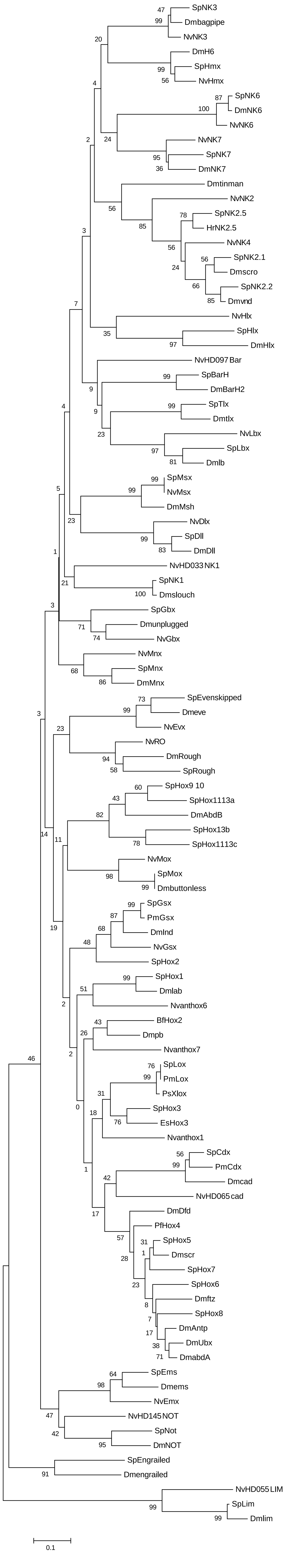

SpNK3
Dmbagpipe
NvNK3
DmH6
SpHmx
NvHmx
SpNK6
DmNK6
NvNK6
NvNK7
SpNK7
DmNK7
Dmtinman
NvNK2
SpNK2.5
HrNK2.5
NvNK4
SpNK2.1
Dmscro
SpNK2.2
Dmvnd
NvHlx
SpHlx
DmHlx
NvHD097 Bar
SpBarH
DmBarH2
SpTlx
Dmtlx
NvLbx
SpLbx
Dmlb
SpMsx
NvMsx
DmMsh
NvDlx
SpDll
DmDll
NvHD033 NK1
SpNK1
Dmslouch
SpGbx
Dmunplugged
NvGbx
NvMnx
SpMnx
DmMnx
SpEvenskipped
Dmeve
NvEvx
NvRO
DmRough
SpRough
SpHox9 10
SpHox1113a
DmAbdB
SpHox13b
SpHox1113c
NvMox
SpMox
Dmbuttonless
SpGsx
PmGsx
DmInd
NvGsx
SpHox2
SpHox1
Dmlab
Nvanthox6
BfHox2
Dmpb
Nvanthox7
SpLox
PmLox
PsXlox
SpHox3
EsHox3
Nvanthox1
SpCdx
PmCdx
Dmcad
NvHD065 cad
DmDfd
PfHox4
SpHox5
Dmscr
SpHox7
SpHox6
Dmftz
SpHox8
DmAntp
DmUbx
DmabdA
SpEms
Dmems
NvEmx
NvHD145 NOT
SpNot
DmNOT
SpEngrailed
Dmengrailed
NvHD055 LIM
SpLim
Dmlim
0.1
